# Genome-wide identification and characterization of miRNAome from tomato (*Solanum lycopersicum*) roots and root-knot nematode (*Meloidogyne incognita*) during susceptible interaction

**DOI:** 10.1371/journal.pone.0175178

**Published:** 2017-04-20

**Authors:** Pritam Kaur, Neha Shukla, Gopal Joshi, Cheeni VijayaKumar, Arun Jagannath, Manu Agarwal, Shailendra Goel, Amar Kumar

**Affiliations:** Department of Botany, University of Delhi, Delhi, India; Tianjin University, CHINA

## Abstract

Root-knot nematodes (RKNs, *Meloidogyne* spp.) are the most damaging plant parasites causing severe losses to crop production. The present study reports genome-wide identification and characterization of both tomato and RKN miRNAs simultaneously from RKN-infected susceptible tomato roots using high-throughput sequencing technique. RNAseq data from 11 small RNA libraries derived from 5 disease development stages identified 281 novel miRNAs of tomato in addition to 52 conserved and 4 variants of conserved miRNAs. Additionally, the same set of RNAseq data identified 38 conserved and 290 novel RKN miRNAs. Both tomato and RKN miRNAs showed differential expression at 5 stages of disease development based on digital expression profiles. In tomato, further validation through qRT-PCR confirmed that majority of miRNAs were significantly upregulated during susceptible response whereas downregulated during resistance response. The predicted targets of 8 conserved and 1 novel miRNAs were validated through 5’RLM-RACE. A negative correlation between expression profiles of a few conserved miRNAs (miR156, miR159, miR164 and miR396) and their targets (*SBP*, *GAMYB-like*, *NAC* and *GRF*1 transcription factor) was confirmed. A novel Sly_miRNA996 also showed a negative correlation with its target *MYB-like* transcription factor. These results indicate that the conserved and novel tomato miRNAs are involved in regulating developmental changes in host root during RKN infection. In RKN, the targets of conserved miRNAs were also predicted and a few of their predicted target genes are known to be involved in nematode parasitism. Further, the potential roles of both tomato and RKN miRNAs have been discussed.

## Introduction

Root-knot nematodes (RKNs, *Meloidogyne* spp.) are the most damaging plant pathogens worldwide. They have a wide host range causing agricultural losses of billions of dollars annually [1,2]. RKNs are sedentary endoparasites that complete their lifecycle within the host roots in 30–45 days. The pre-parasitic second stage juveniles (J2s) invade the plant roots with help of mechanical force and secreted cell wall degrading enzymes through stylet [3]. They initiate the feeding sites known as giant cells that serve as a source of nutrition for developing nematodes [4]. Feeding nematodes become sedentary and undergo through a few round of molting to develop into J3s, J4s and adult females. A large number of secreted effector proteins, which are produced in the esophageal glands, play a crucial role in manipulating host machinery to induce the development of giant cells and to suppress basal host defense responses [5–8]. Several studies have identified repertoire of host genes that are manipulated by effectors during successful infection process. For example, majority of host genes related to defence responses are downregulated and genes involved in cell cycle, cell differentiation, cell wall formation and cytoskeleton organisation are upregulated [9–11].

The role of microRNAs (miRNA) in regulating the expression of genes involved in various biological processes such as growth and development, hormone signalling, abiotic and biotic stress responses have been demonstrated [12–15]. In recent years, the role of plant miRNAs in regulation of genes involved in plant-nematode interaction have also been reported [8,16,17]. The most abundant targets of miRNAs that have been reported with modulated expression during nematode infection are transcription factors. A role of miR396 in modulating the expression of growth regulating factors (*GRF*s) during syncytium formation and maintenance has been demonstrated during cyst nematode infection in *Arabidopsis* [18,19]. Further, Zhao and coworkers [20] reported that miR319/TCP4 module acts as a regulator of jasmonic acid levels upon RKN infection in tomato and thereby, affects the nature of host resistance. Recently, functional role of miR390/TAS3 in regulating *ARF*s at early gall development was studied during *Arabidopsis-*RKN interaction [17]. In animals, role of miRNAs in relation to diseases have been suggested by predicting the miRNA targets computationally in humans, insects and metazoans [21–23]. Recently, cancer associated gender specific miRNAs were identified among human males and females [24]. Additionally, recent studies on identification and characterization of RKN miRNAs suggested their role in nematode development and parasitism [25–27].

In the present study, we have used tomato and RKN (*M*. *incognita*) as a model crop-pathogen system to study the role of miRNAs from both tomato and RKN during disease development. Next generation sequencing approach was employed to identify a population of both tomato and RKN miRNAs from *in vivo* RKN-infected tomato roots at five different disease development stages spanning first day post inoculation (dpi) to 30 dpi. Differential expression of both tomato and RKN miRNAs at various stages indicates their role during disease development. To gain insights into the regulatory roles of miRNAs during RKN infection, in silico target prediction of both conserved miRNAs from tomato and RKN and novel miRNAs from tomato was done and their gene ontology (GO) analysis was also performed. Further, targets of 8 conserved and 1 novel tomato miRNAs were validated through 5’RLM-RACE and correlation in the expression pattern of miRNAs and their targets was determined through qRT-PCR. To the best of our knowledge, this is the first comprehensive study on identification and characterization of miRNAs simultaneously from both tomato and RKN at five different disease development stages that includes early and late stages of RKN infection in tomato roots under soil-grown conditions. Additionally, the role of tomato miRNAs during disease progression and role of RKN miRNAs during its development and parasitism in infected tomato roots have been discussed during tomato-RKN interaction.

## Material and methods

### Nematode culture, plant material, and growth conditions

Disease development study was conducted using RKN, *M*. *incognita*, propagated on susceptible Indian tomato line Pusa Ruby in infection house at Department of Botany, University of Delhi, India. RKN culture was obtained from Prof. Uma Rao’s laboratory, Division of Nematology, Indian Agricultural Research Institute, New Delhi, India. Infection studies were conducted under controlled growth conditions at 22°C with 16 hour light and 8 hour dark photoperiod. Egg masses were dissected manually from infected roots and kept in deionised water at room temperature for hatching into J2s. After 2–3 days, the suspension of J2s was passed through three to four layered kimwipe to separate J2s from unhatched eggs. For miRNA studies, tomato susceptible line, Pusa Ruby and resistant transgenic line that contains *Mi-1* resistance gene were used. Seeds of tomato resistant line were obtained from Prof. Valerie M. Williamson’s laboratory, Department of Nematology, University of California, Davis, USA.

### Stage selection and tissue harvesting

Five-week old seedlings of both susceptible and resistant line were grown in sterile soil and inoculated with 1500 J2s. The plantlets which have not been infected with nematode were treated as control. Collection of tissue was performed in susceptible line for 30 dpi and resistant line for 7 dpi. Plantlets carefully uprooted, roots were quickly washed under cold tap water, blot dried, excised from the plant and snap chilled with liquid nitrogen. Tissue was stored at -80°C until further processing. The root tissue of uninfected plantlets of the same age was also harvested as control samples. Additionally, one infected plant was harvested on each day to study the lifecycle of nematode and progression of the disease through acid fuchsin staining [28]. After staining and clearing, the whole root was observed under the inverted microscope (Carl Zeiss, Gottingen, Germany) and counted the number of nematodes at different development stages. To ensure reproducibility of the time course of disease development, several prior experiments were performed to standardize the count of J2s to be inoculated, substrate required for plant and nematode growth and process for conduction of experiments. Five disease development stages of susceptible response and two stages of resistance response were selected for miRNA study.

### RNA isolation and small RNA library preparation

Root tissue of equal weight of each disease development stage was processed for total RNA isolation using TRI reagent (Sigma-Aldrich, St. Louis, MO, U.S.A.) as per manufacturer’s instructions. The integrity of RNA was checked on 1.2% agarose gel and quantification was done through optical density measurement using Nanovue (GE Healthcare Bio-sciences, Uppsala, Sweden). A total of 11 small RNA libraries were prepared including five infected disease development stages with their corresponding uninfected tissue of same age and a library of five-week old uninfected root tissue of susceptible tomato line. Small RNA libraries from root tissue of each stage were prepared independently using Illumina small RNA sample preparation kit (Illumina, San Diego, CA, U.S.A.) according to manufacturer’s instructions. Briefly, an equal amount of total RNA of each stage was used for sequential ligation of 3’and 5’adapter using T4 RNA ligase. Adapter ligated small RNA was reverse transcribed using SuperScript II Reverse Transcriptase (Invitrogen, Carlsbad, CA, U.S.A.) and 3’ adapter specific RT-primer. cDNA prepared was amplified and product of approximately 147 bp was eluted through 6% polyacrylamide gel. The quality and quantity of cDNA libraries prepared were determined through Agilent 2100 bioanalyzer (Agilent Technologies, Santa Clara, CA, U.S.A.). High-throughput sequencing of libraries was performed using Illumina Hiseq 2000 system at Institute of Genomics and Integrative Biology, Delhi, India, according to manufacturer’s instructions. The supported data was submitted in NCBI’s Gene Expression Omnibus repository under accession number GSE87651 (http://www.ncbi.nlm.nih.gov/geo/query/acc.cgi?acc=GSE87651)

### Bioinformatics analysis of sequencing data

Sequencing data was filtered through a pipeline of UEA sRNA workbench 3.0 with default parameters [29]. Sequences with the desired length between 16–35 nt were extracted and the sequences without 3’ adaptor and those trimmed sequences that did not lie between the specified sequence lengths were discarded. Further, low complexity sequences (sequences containing less than 3 distinct nt), invalid tags with ‘N’ nt, degradation products and other non-small RNAs have been discarded. The fragments of rRNA, tRNA, snRNA or snoRNA were removed after mapping on plant t/rRNAs sequences from “Rfam” (except miRNA), *Arabidopsis* tRNAs from “The Genomic tRNA Database” and plant t/rRNA sequences from “EMBL” release 95.

### Identification of conserved and novel tomato miRNAs

For identification of miRNAs and their precursors, the unique putative small RNA reads were aligned to tomato reference genome dataset obtained from NCBI (Solanaceae Genomics Project Assembly SL2.40) with no mismatch using miRCat pipeline of UEA sRNA tool kit with set parameters [29,30]. The flanking sequences of varying length surrounding the aligned read were extracted from genome and were folded into secondary structures using RNAfold [31]. Further, miRCat trim and analyse the characteristic hairpin structures. The authentic precursors were screened manually to identify the miRNAs based on criteria for annotation of plant miRNAs [32]. To identify conserved miRNAs, mature miRNA sequences were aligned to the known plant miRNAs in miRBase v21 depository [33] by using inbuilt criteria in UEA sRNA workbench. Sequences with perfect homology to any miRNA sequence of miRBASE depository were referred as conserved miRNAs. Additionally, variants of conserved miRNAs were also identified by allowing 2 mismatches. Sequences with ≥ 3 mismatches or no match to already known sequence were referred as novel miRNAs in this study. Further, the conserved miRNAs of tomato were denoted as miR166(i), miR166(ii) and so on, variants of conserved miRNAs were denoted as variant_miR319(i) and the novel miRNAs were denoted as Sly_miRNA (*Solanum lycopersicum*_miRNA)”.

### Prediction of tomato miRNA target genes and GO analysis

To understand the functional role of miRNAs in various biological processes, target genes of miRNAs were predicted by using psRNA Target Analysis Server at default settings [34]. Moreover, transcriptome data used as a reference [35] to predict miRNA targets was generated from RNA isolated from the same set of five disease development stages that were used to obtain the small RNA data.

After prediction, targets were selected for experimental validation. The criteria used for selection of potential targets for validation included 1) inverse correlation in digital expression profile of miRNA and their targets at any stage, 2) putative function in plant development, signal transduction and plant pathogen interaction and 3) low expectation score. Also, all the predicted targets of conserved and novel miRNAs were subjected to GO analysis using SEA tool of AgriGO: A GO analysis toolkit version 1.2 [36]. The enriched GO terms in our dataset with respect to total annotated tomato genes were determined through AgriGO toolkit using Fisher’s exact test at significant P-value < 0.05.

### Validation of target genes of conserved and novel tomato miRNAs through 5’RLM –RACE

The cleavage sites of predicted targets were validated through 5’RLM-RACE. Briefly, the isolation of mRNA/Poly A^+^ RNA from total RNA was performed using PolyATtract mRNA isolation system IV (Promega, Madison, WI, U.S.A.) and 25 ng of mRNA was ligated to the RNA adapter using T4 RNA ligase (Promega). Further, amplification of cleaved products of miRNA target genes was performed using reverse primers specific to target gene and primers complementary to RNA adapter. The PCR amplified products were purified by gel elution, cloned into a pGEMT Easy vector (Promega) and sequenced. The sequencing results were analysed to map the cleavage sites. Primers used in 5’ RLM-RACE are detailed in the S1 Table.

### Expression profiling of tomato miRNAs and their targets through qRT-PCR

The variation in expression of conserved and novel miRNAs was confirmed by performing qRT-PCR across four stages (stage 1, 2, 3 and 5) of susceptible response and two stages (stage 1 and 2) of resistance response. The tissue of corresponding uninfected stage was considered as control to reduce the biasness due to the root development. Total RNA (10 ug) was used for DNAase treatment with Deoxyribonuclease I (New England Biolabs, Ipswich, MA, U.S.A.) followed by phenol-chloroform purification. 2 ug of purified DNAase-treated RNA was polyadenylated using *E*. *coli* Poly-A polymerase I provided in Poly-A tailing kit (Ambion, Austin, TX, U.S.A.) according to manufacturer’s instructions. The polyadenylated product was purified and reverse transcribed using a poly-T adapter and SuperScript Reverse Transcriptase III (Invitrogen). Further, expression of miRNAs was measured through Taqman probe based qRT- PCR method (Roche, Mannheim, Germany). The qRT-PCR for expression analysis of miRNAs was performed using miRNA specific forward primer (0.5 μM), universal reverse primer (0.5 μM), TaqMan probe complementary to the poly-T adapter (0.2 μM) with LightCycler^®^ 480 Probe Master Mix. 18S rRNA was used as internal reference control. The absolute quantification of 18S rRNA gene was performed to normalize the copy number in each sample to eliminate any biasness caused during RNA quantification and poly-A tailing. The reaction cycling conditions were as follows—denaturation and hot start activation of the enzyme at 95°C for 1 min then 40 cycles at 95°C for 10 s followed at 60°C for 40 s for amplification. For miRNA target gene expression profiling, DNase-treated RNA was reverse transcribed using iScript reverse transcription kit (BioRad, Hercules, U.S.A.) as per manufacturer’s instructions. Expression profile of target genes was determined using gene-specific primers (0.5 μM) and SYBR green I master mix (Roche) in CFX connect Real-Time PCR detection system (BioRad). Open reading frame that showed a uniform expression in all the transcriptome libraries of same five disease development stages generated in our lab was chosen as endogenous control [35]. The expression profiling of miRNAs and their target genes was done with two technical replicates for each of three biological replicates in CFX connect Real-Time PCR detection system (BioRad). Relative expression was determined by calculating fold change in infected sample relative to uninfected sample using delta delta Ct method [37]. Additionally, student’s t-test (*P* < 0.05) was performed to determine the significant difference in expression of miRNAs between infected and corresponding uninfected stages. Primers used in qRT-PCR are detailed in the S1 Table.

### Identification of conserved and novel RKN miRNAs

Unique reads obtained after filtration of sequencing data were mapped on RKN reference genome obtained from NCBI (whole genome shotgun sequence assembly, strain Morelos) with no mismatch for identification of miRNAs through miRCat [29]. The flanking sequences around aligned reads of various lengths were extracted from the reference genome and hairpin structures were prepared through RNAfold [31] and analyzed for the identification of potential miRNA precursor using UEA sRNA toolkit [29]. For the identification of conserved miRNAs, predicted RKN miRNA sequences were aligned with known miRNA sequences in the miRBASE v21 depository [33]. The criteria used for identification of conserved miRNAs was same seed sequence (2–7 nt from 5’ end of miRNA sequence) and 80% homology within the mature sequence (21–24 nt) with the known miRNAs in miRBASE v21 depository. The remaining miRNAs with less than 80% homology to the already known miRNA sequences were referred as novel miRNAs. In this study, the conserved miRNAs of RKN were denoted as miR-100_1, miR-100_2 and so on and the novel miRNAs were denoted as min_miRNA (*Meloidogyne incognita*_miRNA).

### Prediction of conserved RKN miRNA targets and GO enrichment analysis

We have predicted the targets of nematode conserved miRNAs through miRanda software [23, 38] at default parameters using available 3’UTR sequences of RKN genes obtained from WormBase (http://www.wormbase.org). Targets of 37 conserved miRNAs were predicted on the basis of strict complementarity of seed sequence (2–7 nt) at 5’ end of the miRNA. Further, GO enrichment analysis of all miRNA targets was performed through GeneMerge v1.4 and considered Bonferroni corrected P-value < 0.05 [39].

## Results

### Disease development

**Fig 1:**
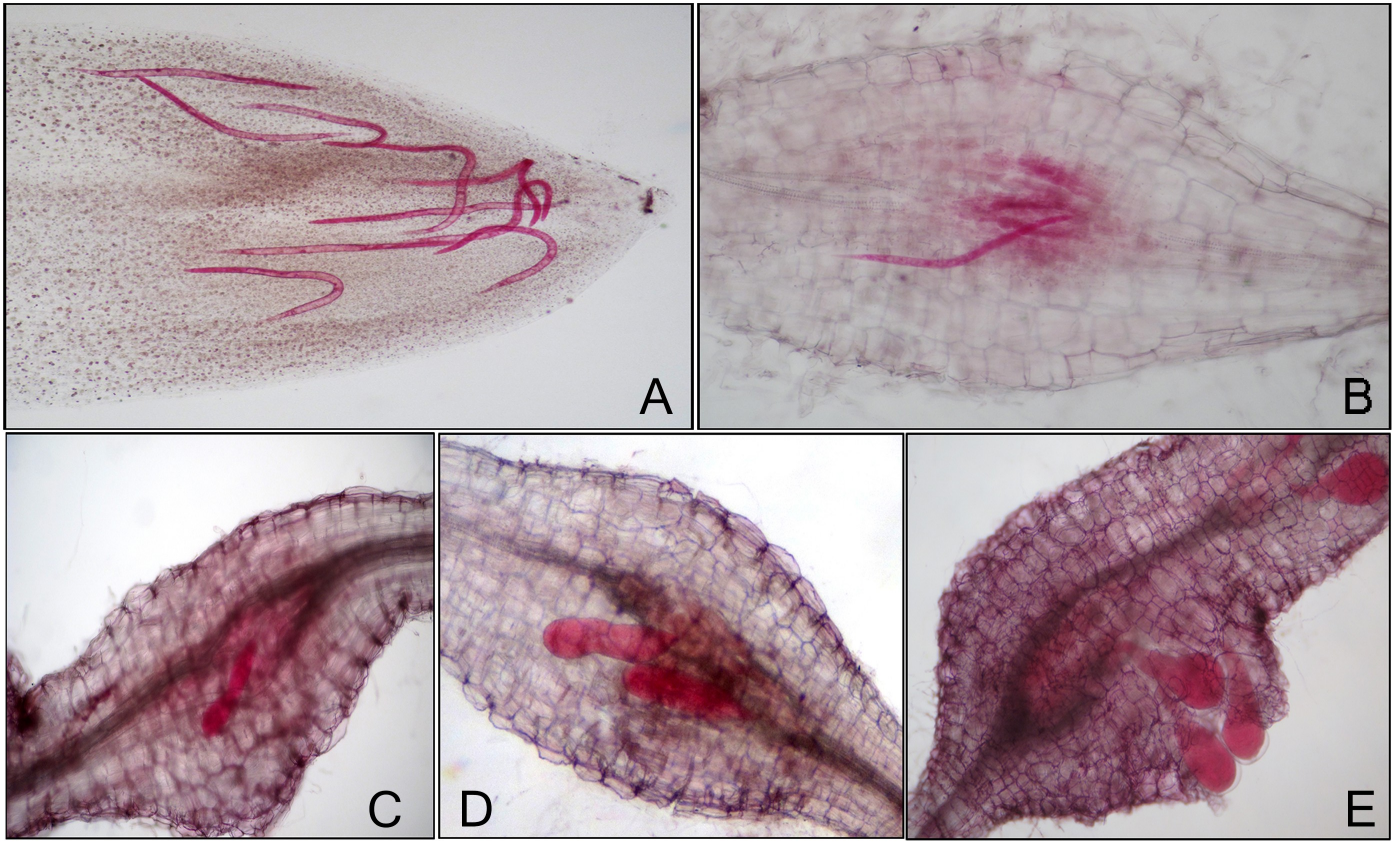
Different stages of disease and RKN development observed in infected tomato roots of susceptible line through acid fuchsin staining. **(A)** Invasion of J2s/ initiation of feeding sites (Stage 1). **(B)** Parasitic J2s/ formation of feeding sites (Stage 2). **(C)** Feeding J2s and J3s/ expansion of feeding sites (Stage 3). **(D)** J4s/ maintenance of feeding sites (Stage 4). **(E)** J4s and females/ maintenance of feeding sites (Stage 5).

Disease progression was studied to define the stages in RKN-infected tomato roots of susceptible and resistant lines grown in soil conditions. In RKN-infected susceptible line, the complete life cycle was divided into five stages on the basis of number of J2s invading in the tomato roots, formation of giant cells and root-knots, transition in different development stages of nematode and progression of disease in the roots. We classified the root tissues pooled from 1, 2 and 3 dpi as stage 1 (invasion of J2s/ initiation of feeding sites); 5, 6 and 7 dpi as stage 2 (parasitic J2s/ formation of feeding sites); 13, 14 and 15 dpi as stage 3 (feeding J2s and J3s/ expansion of feeding sites); 18, 19 and 20 dpi as stage 4 (J4s/ maintenance of feeding sites) and 26, 27 and 28 dpi as stage 5 (J4s and females/ maintenance of feeding sites). These five disease development stages selected for further study are depicted in Fig 1. In RKN-infected resistant line that possesses *Mi-1* gene, only two stages of disease development corresponding to first two stages of susceptible response were considered for the study.

### Analysis of small RNA sequencing data

High throughput sequencing of 11 small RNA libraries prepared from 5 stages of disease development from susceptible line yielded a total of 517 million raw reads and 36 million unique reads. The sequencing data set of these small RNA libraries was pooled and filtered using UEA sRNA workbench version 3.0 [29]. In total, 221 million raw reads (42.7%) and 20 million unique reads (56.5%) were accounted as putative small RNA reads of 16–35 nucleotide (nt) length and were used for further analysis (Table 1).

**Table 1:** Elimination summary showing the abundance of reads obtained from all the libraries of susceptible interaction investigated.

Type of reads	Total raw reads	Unique reads
Raw reads	517948240 (100%)	36846758 (100%)
Sequence after 3’ adapter removal	504652329 (97.4%)	25805141 (70%)
Filter by sequence length (<16nt and >35nt)	170832869 (32.9%)	15789097(42.8%)
Filter low-complexity sequences	5352 (0.001%)	3861 (0.01%)
Filter invalid sequences	129588 (0.02%)	63121 (0.17%)
Filter by t/rRNA (matches out)	125801908 (24%)	166981 (0.45%)
Putative small RNA (sRNA) population	221178523 (42.7%)	20823698 (56.5%)

In our sequencing dataset, majority of small RNA reads were 20–24 nt in length suggesting the role of dicer proteins in small RNA production [40]. Among raw reads, 21 nt length reads were abundant whereas among unique reads, 24 nt length reads were higher followed by reads of 23 nt, 22 nt and 21 nt length (S1 Fig). The 23–24 nt length reads belong to class of heterochromatic siRNAs and are reported as most abundant reads during small RNA sequencing in other flowering plants [40]. The 23 nt reads might be generated due to degradation of 24 nt fragments. These results are consistent with the previous studies on Citrus, Soybean and Potato small RNAs [41–43]. For further analysis, we considered the characteristic DCL1 processed 21 nt length reads and also 20 and 22 nt length reads that could have been produced due to inappropriate processing by DCL1.

### Identification of conserved and novel tomato miRNAs

After filtration, all unique sequences were mapped on tomato reference genome for identification of miRNAs using UEA sRNA toolkit plant version miRCat pipeline with no mismatch [29,30]. The flanking regions of varied length on both sides of mapped sequence were extracted from the genome and secondary structures of these regions were prepared through RNAfold. The secondary structures were trimmed and analysed through miRCat to identify potential precursors. These potential precursors were screened for identification of candidate miRNA following the criteria for miRNA annotation as described by Meyers et al. [32]. Out of total 5184 predicted small RNAs, 1775 candidate miRNAs were of 20–22 nt in length. Among these, 337 miRNAs with more than 10 reads in all the libraries were further characterized. Fifty-two conserved miRNAs belonging to 23 miRNA families (with prefect homology), 4 variants of conserved miRNAs (with ≤ 2 mismatches) and 281 novel miRNAs (with ≥ 3 mismatches) were identified on the basis of miRBASE v21 depository. Out of 52 conserved and 4 variants of conserved miRNAs, mature sequences of 42 conserved and all variants of conserved miRNAs were predicted from both the opposite arms of stem-loop precursors (S2 Table). Among 281 predicted novel miRNAs, mature sequences of only 60 were identified from both the opposite arms of stem-loop structure and here after referred as novel miRNAs (S3 Table). For the remaining 221 miRNAs, only one mature sequence was identified, which might be due to degradation of other sequence during biogenesis of miRNA [13] and they were referred as candidate novel miRNAs (S4 Table). The average of adjusted minimum free energy (AMFE) of conserved miRNA precursors was -45 kcal/mol and novel miRNA precursors was -40.7kcal/mol, which is in accordance to the known free folding energies of miRNA precursors in other plant species [44]. The stem-loop secondary structure of a few novel miRNAs predicted through Mfold web server is shown in S2A Fig.

### Genomic distribution of conserved and novel tomato miRNAs

Most of the plant *MIR* genes are transcribed from intergenic regions of the genome [45]. In our study, distribution of pre-miRNA loci of 337 miRNAs was investigated among intergenic and genic regions of tomato genome (Solanaceae genome network). Out of 337 miRNAs, 296 were transcribed from intergenic regions and 41 from genic regions (S3 Fig). Among the miRNAs that are transcribed from genic regions, 27 were encoded by introns, 12 from exons and 2 from both introns and exons.

**Fig 2:**
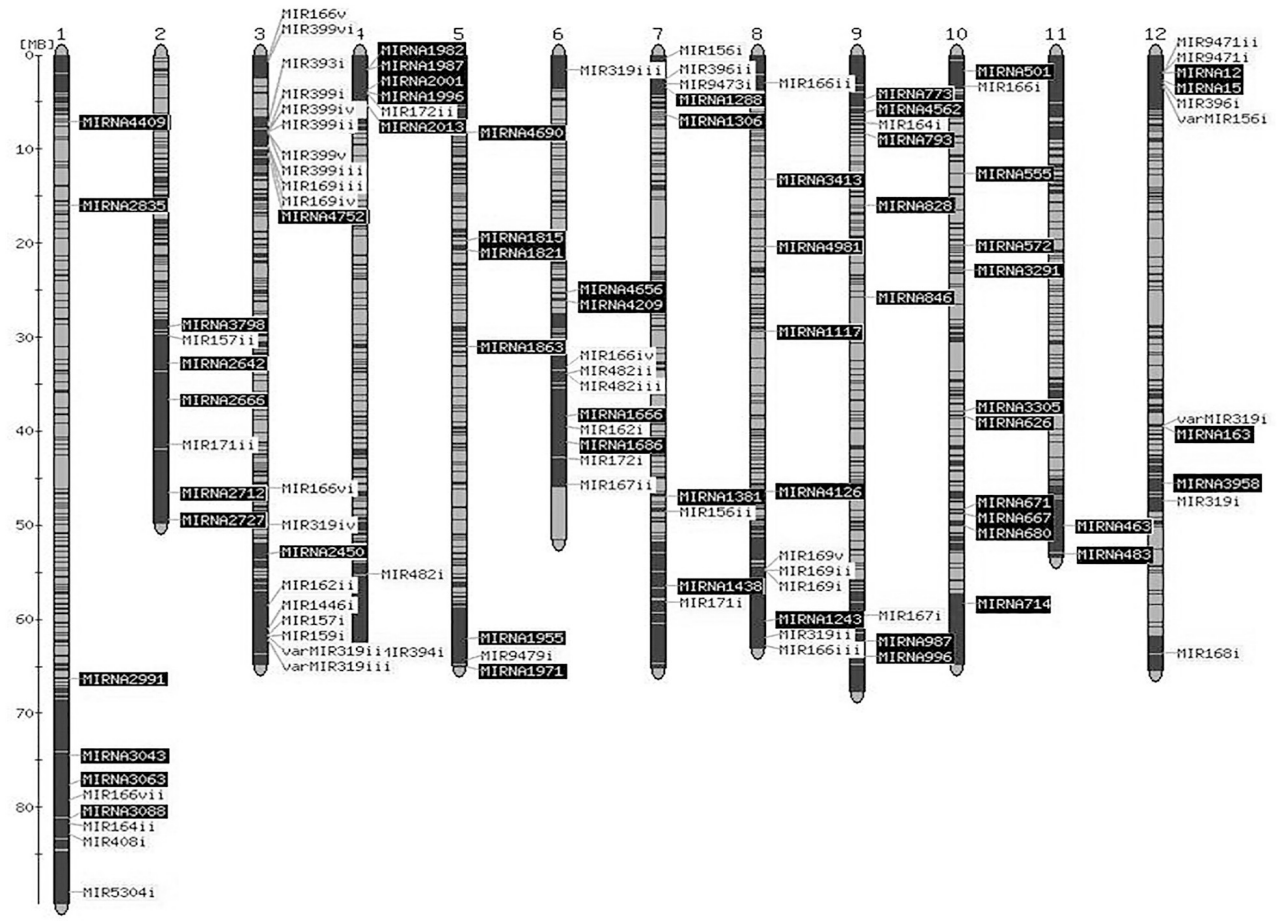
Chromosomal distribution of predicted miRNA precursors of tomato miRNAs. The precursors of conserved (MIR), variants of conserved (varMIR) and novel (MIRNA) miRNAs were mapped onto tomato chromosomes using sequenced based map (Mb units) that shows the positions of Kazusa and SolCAP markers on the genome. The loci of pre-miRNAs of conserved and variants of conserved miRNAs are highlighted in light color and the loci of pre-miRNAs of novel miRNAs are highlighted in dark colour.

Chromosomal distribution of pre-miRNA loci of predicted 52 conserved miRNAs, 4 variants of conserved miRNAs and 60 novel miRNAs was investigated (Fig 2). We found random distribution of these miRNAs across twelve chromosomes when loci of pre-miRNAs were overlayed on a map that shows the position of “kazusa and SolCAP markers” (Solanaceae genome network). In plant genome, such random distribution of miRNAs suggests evolution of genes through different duplication events and inversion followed by chromosomal rearrangements, amplification and loss of genes [46]. The density of miRNAs was highest on chromosome 3 including 18 conserved and 2 novel miRNAs and least on chromosome 11 including no conserved and 2 novel miRNAs (Fig 2).

Interestingly, 5 members of miR399 family were present in close proximity on chromosome 3 (Fig 2). These members shared same miRNA mature sequence, digital expression profile and predicted targets (S5 and S8 Tables). However, sixth member of miR399 family was located at a different position on the same chromosome and has different mature sequence, expression profile and predicted targets. Also, 3 members of miR319 family with the same mature sequence and expression profile were located on different chromosomes (chromosome 6, 8 and 12). The fourth member, miR319(iv), differing in one nt at 3’end along with different expression profile was located on chromosome 3 (Fig 2). In miR166 family, seven members with the same mature sequence, expression profile and predicted targets, but having different precursor sequences, were distributed on different chromosomes (chromosome 1, 3, 6, 8 and 10). Furthermore, 17 novel miRNAs having same mature sequence, expression profile and predicted targets (S6 and S9 Tables), were located on seven different chromosomes (chromosome 1, 2, 7, 9, 10, 11 and 12; Fig 2).

### Digital expression profiling of tomato miRNAs

Normalization of reads across all the samples was done as transcripts per million (TPM) and variation in the expression of conserved, variants of conserved, novel and candidate novel miRNAs across all stages was estimated (S5–S7 Tables). For further analysis, only conserved, variants of conserved and novel miRNAs were considered. Out of 52 conserved miRNAs, miR166 family and miR396(i) were highly expressed (total TPM value > 10,000). Amongst these, miR166 family was abundantly expressed (total TPM value > 4,00,000). In contrast, a low digital expression was observed for miR1446, miR167, miR169, miR171, miR393, miR394, miR399, miR408, miR5304, miR9473 and miR9479 families (total TPM value < 100). Expression of all the variants of conserved miRNAs was also low (total TPM value < 100).

Similar to previous reports [42,47], we observed a lower expression of novel miRNAs than that of conserved miRNAs. For instance, the highly expressed novel miRNA, Sly_miRNA1117 has total TPM value of 153 followed by Sly_miRNA996 (total TPM value of 147). Whereas, the low expression was observed for Sly_miRNA1863 (total TPM value 11.63) and Sly_miRNA463 (total TPM value 11.13). A few highly expressed novel miRNAs that qualified the criteria for plant miRNA annotation [32], were selected for validation.

### Expression profiling of conserved and novel tomato miRNAs through qRT-PCR

**Fig 3:**
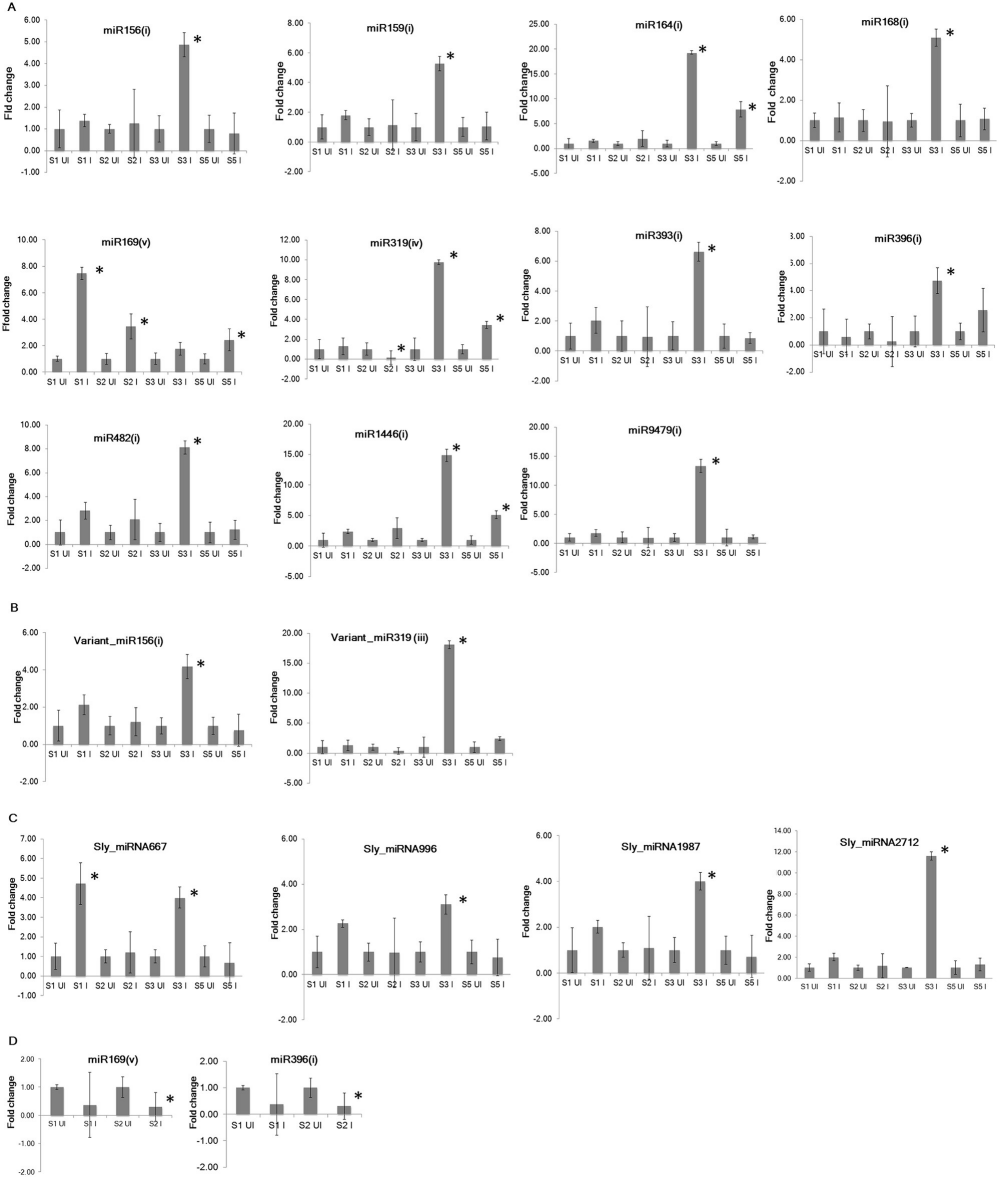
Expression analysis of tomato miRNAs through qRT-PCR at four stages of disease development during susceptible response (A-C) and at two stages of disease development during resistance response (D). To normalize the expression level, 18S rRNA was selected as internal control. All the experiments were conducted using two technical replicates for each of three biological replicates. Fold change was calculated through delta delta Ct method that represents the change in expression level in the infected sample relative to uninfected control sample. Data is the average of three biological replicates ± standard error of the mean. The student’s t-test (P < 0.05) was performed to determine significant difference in miRNA expression between uninfected and infected sample. The significant difference (P < 0.05) obtained for an infected stage is marked with * in the figure. S1-Stage 1, S2-Stage 2, S3-Stage 3, S5-Stage 5, UI-Uninfected sample, I-Infected sample.

Expression profiles of 11 conserved, 2 variants of conserved and 4 novel miRNAs were determined through qRT-PCR. The qRT-PCR results confirmed that majority of conserved and novel miRNAs were upregulated at different disease development stages during susceptible response. Most of the conserved and novel miRNAs were significantly upregulated (*P* < 0.05) at stage 3 (Fig 3). Among all validated miRNAs, the highest expression at stage 3 was observed for miR164(i) followed by variant_miR319(iii), miR9479(i) and miR319(iv). A few miRNAs including miR393(i), miR482(i), miR1446(i) and variant_miR156(i) were also upregulated at stage 1 (Fig 3A and 3B), whereas, miR164(i), miR319(iv) and miR1446(i) were upregulated (*P* < 0.05) at stage 5 (Fig 3A). Further, miR319(iv) was downregulated (*P* < 0.05) at stage 2. The only miRNA, miR169(v), was upregulated (*P* < 0.05) at stages 1, 2 and 5 with the highest expression at stage 1 (Fig 3A). Further, differential expression of 4 novel miRNAs including, Sly_miRNA667, Sly_miRNA996, Sly_miRNA1987 and Sly_miRNA2712 was observed (Fig 3C). Among all 4 upregulated miRNAs, only Sly_miRNA667 was significantly upregulated (*P* < 0.05) at stage 1. In contrast to susceptible response, most of the miRNAs were downregulated during resistance response. For example, conserved miR396(i) and miR169(v) were downregulated (*P* < 0.05) at stage 2 (Fig 3D).

### Prediction of conserved and novel tomato miRNA targets, GO analysis and validation through 5’RLM-RACE

Putative targets were predicted for 52 conserved, 4 variants of conserved and 59 novel miRNAs (S8 and S9 Tables). The predicted targets of conserved miRNAs were the same as was observed for other plant miRNAs with majority of them being transcription factors. For example, known targets, *SBP*, *NAC*, *GRAS*, *HB*, *GRF*, *GAMYB-like* and *TCP24* transcription factors were predicted for miR156(i), miR164(i), miR171(i), miR166(i), miR396(i), miR159(i) and miR319(i), respectively in our study. In case of novel miRNAs, Sly_miRNA2712 was predicted to target peroxidase. Sly_miRNA667 was predicted to target transmembrane receptor serine/threonine kinase protein and Sly_miRNA996 was predicted to target *MYB-like* transcription factor.

**Fig 4:**
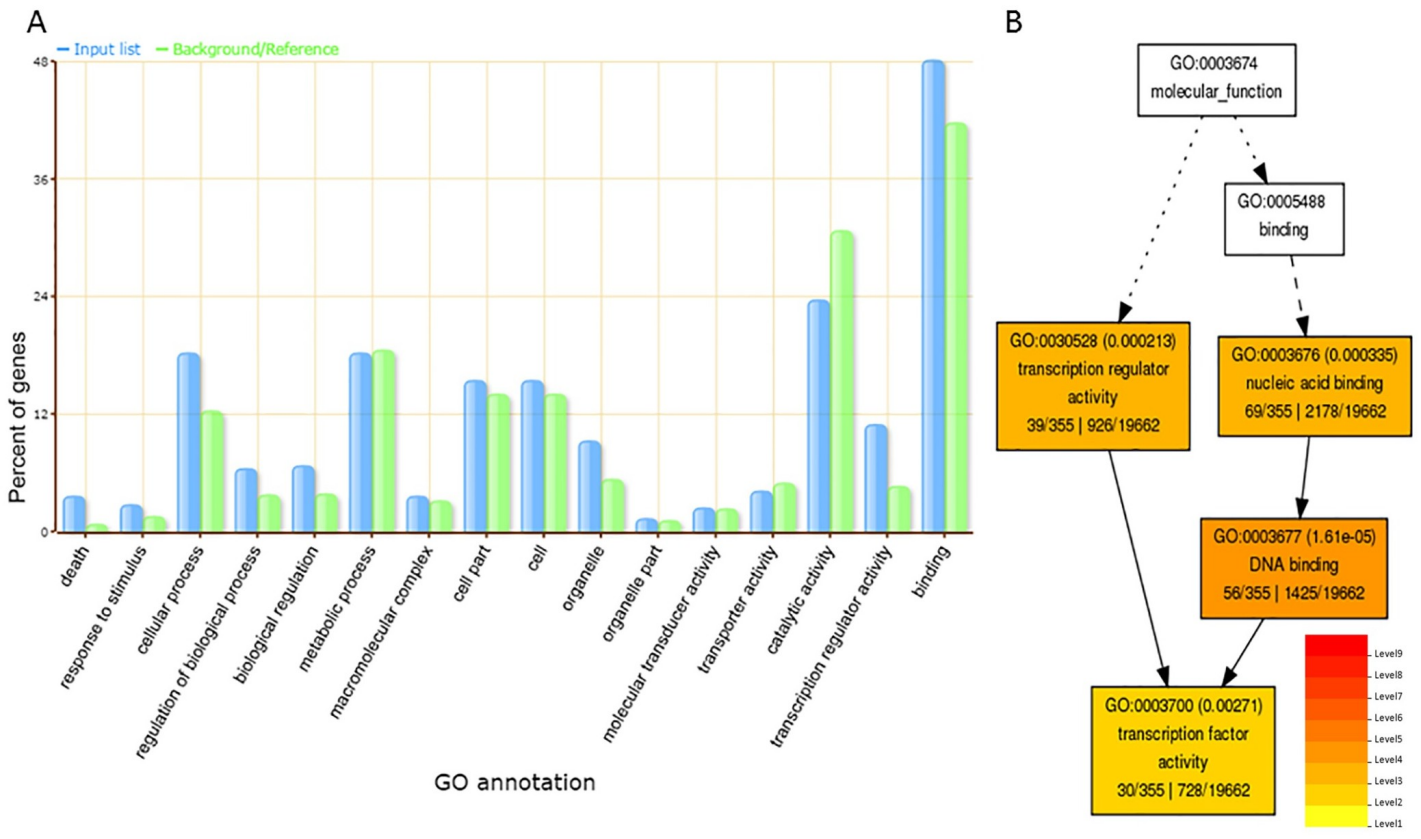
GO analysis of targets of conserved, variants of conserved and novel tomato miRNAs using singular enrichment analysis tool (SEA) of AgriGO toolkit. (**A)** The percentage of genes mapped under different GO terms is represented through a bar graph. Blue bars indicate percentage of enriched miRNA target genes in different GO terms and green bars indicate percentage of total annotated tomato genes mapping in different GO terms. (**B)** Enriched GO terms in molecular function category are represented in hierarchical tree graph.

GO annotation and enrichment analysis was performed for all the predicted targets of conserved, variants of conserved and novel miRNAs to investigate their potential functions using AgriGO v1.2 [36]. A total of 19,662 annotated tomato genes in AgriGO database were used for GO singular enrichment analysis (SEA) of 516 putative miRNA targets. Out of these target genes, GO of 355 genes was achieved, which were categorized into cellular component (GO:0005575), biological processes (GO:0008150) and molecular functions (GO:0003674) (S10 Table). The enriched target genes were mapped under significant GO terms of each category (Fig 4A). A high percentage of genes encoding transcription factors were enriched under GO term, transcription factor activity (GO:0003700) of molecular functions (Fig 4B). The majority of genes associated with biological processes and cellular component were enriched in GO terms, cellular processes (GO:0009987) and intracellular membrane-bound organelle (GO:0043231), respectively. While other enriched GO terms under biological processes include death (GO:0016265) and defense responses (GO:0006952).

**Fig 5:**
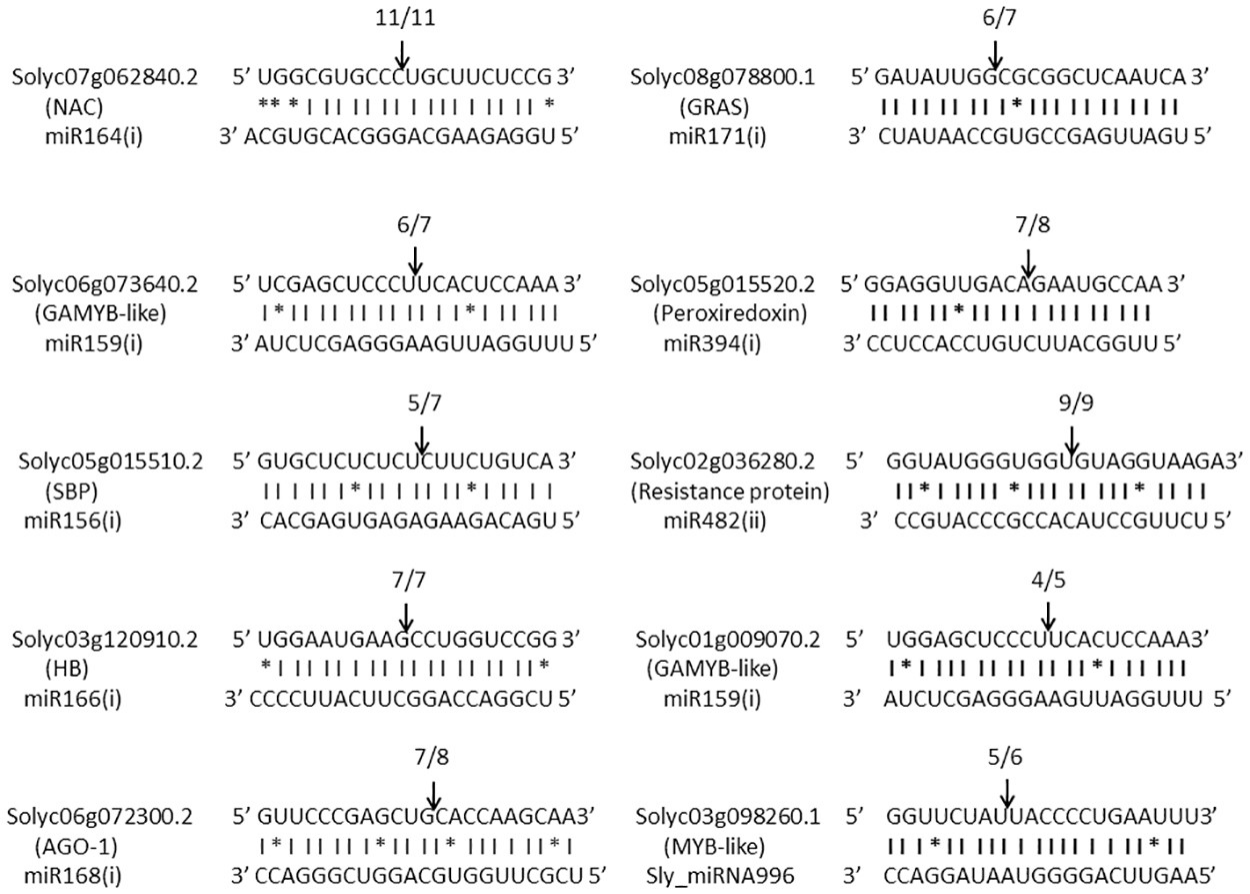
Mapping of amplified cDNA cleaved products obtained from 5’RLM-RACE. Using psRNAtarget Analysis server, conserved miRNAs including, miR164(i), miR171(i), miR159(i), miR394(i), miR156(i), miR482(ii), miR166(i), and miR168(i) were predicted to target genes including *NAC*, *GRAS*, *GAMYB-like*, Peroxiredoxin, *SBP*, Resistance protein, *HB* and *AGO-1*, respectively. A novel miRNA, Sly_miRNA996 was predicted to target *MYB-like* transcription factor gene. The cleaved products were amplified through 5’RLM-RACE, cloned in pGEMT vector and mapped. Cleavage sites are depicted with arrows and number of positive clones obtained out of total clones is mentioned above the arrows.

The targets of 8 conserved miRNAs and 1 novel miRNA have been validated through 5’ RLM-RACE (Fig 5). The sequencing results of cloned 5’RLM-RACE cleavage products revealed that cleavage site of *SBP* (Solyc05g015510.2) and peroxiredoxin (Solyc05g015520.2) lies between 9^th^ and 10^th^ base from 5’ end pairing of miR156(i) and miR394(i), respectively (Fig 5). The cleavage site of genes including *NAC* (Solyc07g062840.2), *HB* (Solyc03g120910.2), *AGO1* (Solyc06g072300.2), Resistance protein (Solyc02g036280.2) and *GAMYB-like* (Solyc06g073640.2, Solyc01g009070.2) was deciphered to lie between 10^th^ and 11^th^ base from 5’ end pairing of miR164(i), miR166(i), miR168(i), miR482(ii) and miR159(i), respectively (Fig 5). The transcription factor gene, *GRAS* (Solyc08g078800.1) was shown to be cleaved by miR171(i) between 13^th^ and 14^th^ base from 5’ end binding of miRNA. We also attempted to validate the targets of four novel miRNAs, however, the cleavage site at expected position was confirmed only for *MYB-like* transcription factor gene (Solyc03g098260.1), which is the target of Sly_miRNA996. The cleavage site of Sly_miRNA996 on its target lies between 13^th^ and 14^th^ base from 5’ end of miRNA binding which is similar to the cleavage position of target gene (*GRAS*) of miR171(i) (Fig 5).

### Correlation between expression of tomato miRNAs and their target genes based on qRT-PCR analysis

**Fig 6:**
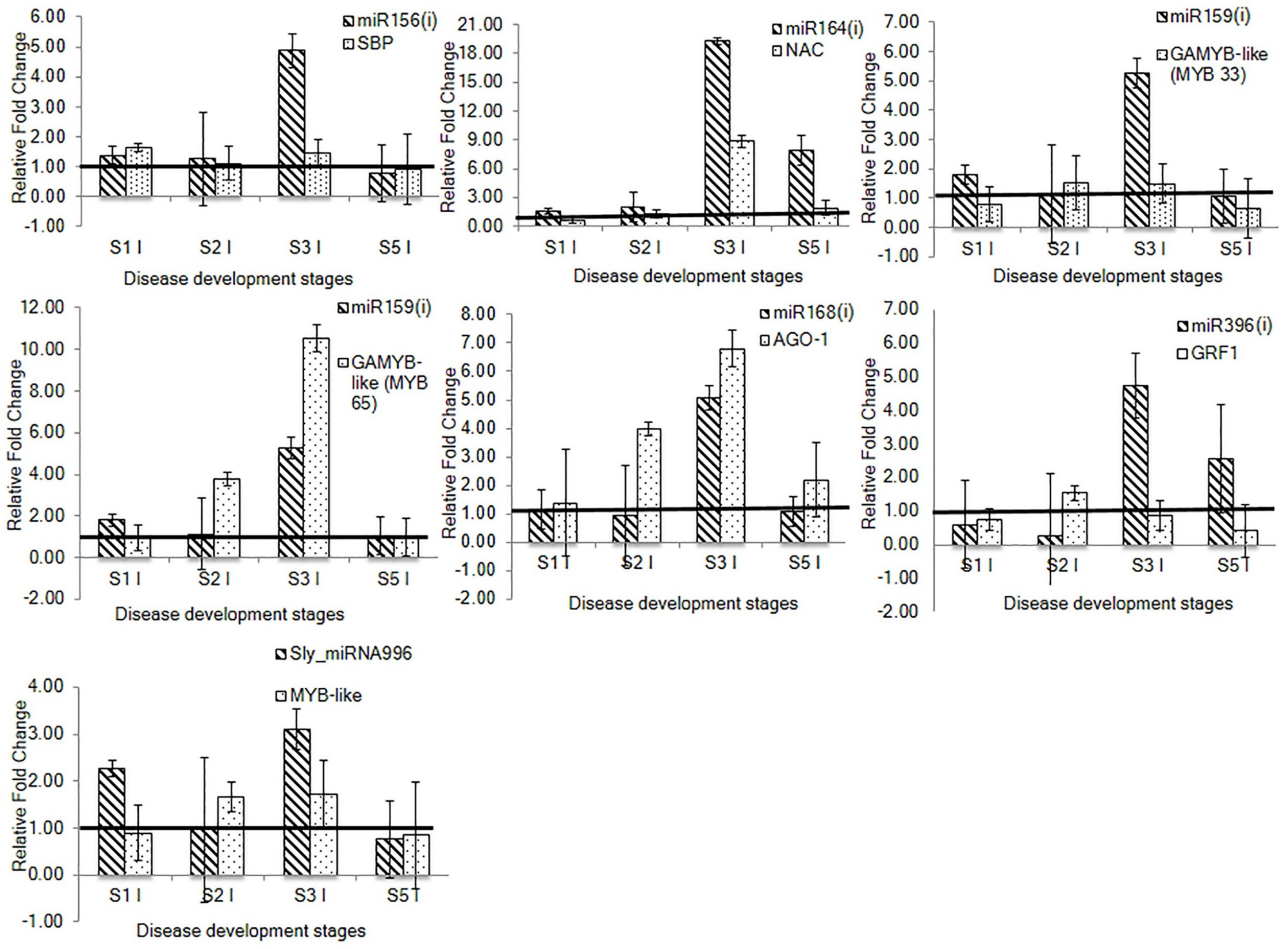
Relative expression analysis of selected conserved and novel tomato miRNAs and their targets through qRT-PCR at four stages of disease development during tomato-RKN susceptible interaction. The correlation in expression profile was deciphered between conserved miRNAs including, miR156(i), miR164(i), miR159(i), miR168(i) and miR396(i) and their target genes, *SBP*, *NAC*, *GAMYB-like* (*MYB33* and *MYB65*), *AGO1* and *GRF1*, respectively. The correlation in expression profile of novel miRNA, Sly_miRNA996 with its target, *MYB-like* transcription factor gene was also determined. For qRT-PCR analysis, two technical replicates for each of three biological replicates were used. Fold change was calculated through delta delta Ct method that represents the change in expression level of miRNA and target gene in the infected sample relative to the uninfected control. The fold change of uninfected sample of each stage was taken as 1 and presented with solid line. The data is presented as the mean of three biological replicates ±standard error of the mean. S1-Stage 1, S2-Stage 2, S3-Stage 3, S5-Stage 5, UI-Uninfected sample, I-Infected sample.

The miRNA targets validated through 5’RLM-RACE, were selected for expression analysis through qRT-PCR. A negative coorelation between miRNAs and their target genes was determined at different stages of disease development during which miRNAs were upregulated and their targets were downregulated. For example, miR164(i) and *NAC* transcription factor gene showed negative correlation at stages 3 and 5. While a negative correlation between miR156(i) and *SBP* was observed at stage 3 only (Fig 6). Interestingly, miR159(i) cleaved two *GAMYB-like* transcription factor genes (*MYB33* and *MYB65*) but a negative correlation was observed with only *MYB33* gene at stage 3. Although, miR159(i) showed inverse correlation with its other target gene, *MYB65* at stage 3, the expression of target gene was higher than miR159(i). Similarly, in the case of miR168(i) and *AGO1*, the expression of target gene was higher than the expression of miRNA at different stages (Fig 6).

We have also studied the correlation in expression pattern between miR396 and *GRFs* based on previous reports [18–20]. In concordance with previous studies, we observed a negative correlation in the expression profiles of miR396(i) and *GRF1* gene. At later stage (stage 3), miR396(i) was upregulated and *GRF1* was downregulated (Fig 6). Since miR396(i) also targets *GRF4* gene, Zhao et al. [20] showed change in expression of GRF4 during tomato-RKN interaction, therefore we have also looked the expression of *GRF4*, but no significant modulation in its expression was observed. Additionally, a novel Sly_miRNA996 showed negative correlation with its target, *MYB-like* transcription factor gene (an unknown protein of uncharacterised function) at stages 1 and 3 (Fig 6).

### Identification of conserved and novel RKN miRNAs

The genome sequence of RKN was used to identify miRNAs through UEA sRNA workbench version 3.0 [29]. Only 0.76% of small RNA reads were mapped to RKN genome sequence with no mismatches. In total, 328 RKN miRNAs of 21–24 nt length were identified. Further, 24 miRNAs belonging to 14 miRNA families (miR-1, miR-9, miR-34, miR-50, miR-72, miR-92, miR-100, miR-124, miR-252, miR-277, miR-279, miR-7904, let-7 and lin-4) with ≤ 2 mismatches with miRBASE v21 depository were identified. Seven conserved miRNAs belonging to 4 miRNA families (miR-2, miR-57, miR-100 and miR-993) with 3 mismatches and additional 7 miRNAs belonging to 5 miRNA families (miR-39, miR-58, miR-76, miR-86 and miR-184) were identified on the basis of 80% homology with miRBASE v21 depository. Therefore, a total of 38 conserved RKN miRNAs belonging to 22 miRNA families were identified, out of which mature sequences were derived from both the arms of 24 miRNAs (S11 Table). Out of remaining 290 miRNAs, mature sequences were derived from both the arms of only 106 miRNAs that were referred as novel miRNAs and rest 184 miRNAs were referred as candidate novel miRNAs (S12 and S13 Tables). The average of AFME of conserved miRNAs was -37.38 kcal and of novel miRNAs was -52.21 kcal, suggesting the formation of highly stable secondary stem-loop structure of precursor miRNAs. The characteristic structures of a few novel miRNAs were prepared from Mfold web server (S2B Fig).

### Digital expression profile of conserved and novel RKN miRNAs

The conserved and novel miRNAs with ≥ 10 total reads across all libraries were analyzed. Among conserved miRNAs, miR-100_3 has high level of expression (total TPM value > 3000) followed by miR-92_1 (total TPM value >1000). A few miRNAs showed a gradual increase in expression across all five stages of infection with highest expression at stages 4 and 5. These include miR-2_1, miR-57_1, miR-58_1, miR-100_1 and lin-4 (total TPM value 400–800). The low expression level was observed for miR-9_1, miR-39_1, miR-72_1, miR-86_1, miR-124_2, miR-184_1, miR-993_1 and miR-7904_1 (total TPM value < 100) in all the stages of infection (S14 Table).

The expression pattern of novel miRNAs was similar to conserved miRNAs, but the level of expression was low (S15 Table). Among novel miRNAs, min_miRNA15 has a high level of expression (total TPM > 700) followed by min_miRNA160 (total TPM > 500). Whereas a low level of expression was observed for min_miRNA6, min_miRNA105, min_miRNA128, min_miRNA206, min_miRNA306 and min_miRNA312 (total TPM value < 80).

### Conservation of RKN miRNAs

The extent of conservation of miRNAs was investigated among a plant parasitic nematode (*M*. *incognita*), two free-living nematodes (*Caenorhabditis elegans* and *Panagrellus redivivus*), an animal parasitic nematode (*Ascaris suum*) and a human parasitic nematode (*Brugia malayi*) (S16 Table). The mature sequences of conserved miRNAs identified in our study were compared to the conserved miRNA sequences of these nematodes retrieved from miRBASE v21 depository. The conservation was studied on the basis of miRNAs with same seed sequences (2–7 nt from 5’ end of miRNA) and at least 80% homology within the mature sequence (21–24 nt). Out of 23 unique conserved RKN miRNAs identified in this study, 7 were conserved in *C*. *elegans*, 17 in *P*. *redivivus*, 12 in *B*. *malayi* and 19 in *A*. *suum*. Interestingly, 6 miRNAs (miR-34_1, miR-50_1, miR-72_1, miR-124_2, miR-252_1 and let-7) were conserved across all the nematode species investigated in this study. Further, miR-2_1, miR-57_1, miR-92_1, miR-124_1, miR-184_1 and miR-279_1 were identified in the genomes of parasitic nematodes (*M*. *incognita*, *A*. *summ*, and *B*. *malayi)* only. A few RKN miRNAs including miR-39_1, miR-58_1, miR-277_1 and miR-7904_1 were conserved in *P*. *redivivus* however, absent from *A*. *summ* and *B*. *malayi*. Also, a few *M*. *incognita* miRNAs including miR-9_1, miR-86_1 and miR-100_1 were conserved in *P*. *redivivus* and parasitic nematodes, but absent from *C*. *elegans*.

The conserved miRNAs, miR-1_1, miR-34_1 and miR-124_1 identified as RKN miRNAs in our study have not been reported previously. Moreover, among these, miR-124_1 was conserved in only the genome of *A*. *suum* (S4A Fig). Three additional conserved miRNAs (miR-71, miR-81, miR-228) were identified when mapped on *C*. *elegans* genome [48] with no mismatches. All three miRNAs also showed homology with known miRNAs in other nematode species (S4B Fig). However, these miRNA sequences could not be mapped on the available RKN genome database, even if the stringency was reduced to 80% homology within the mature sequence.

### Prediction of conserved RKN miRNA targets and GO analysis

In mammals, miRNAs guide the post-transcriptional gene silencing of mRNAs by complementary base-pairing at 3’ untranslated region (3’ UTR). We predicted the targets of conserved RKN miRNAs using available 3’ UTR sequences of RKN genes based on strict seed sequence complementarity criteria. A total of 4886 targets of conserved miRNAs were predicted (S17 Table), of which a few were predicted to target several effectors. For example, the predicted targets of miR-7904_1 were chorismate mutase, 14-3-3 peptide, cysteine peptidase, glycoside hydrolase and C-type lectin. Also, miR-72_1 was predicted to target nematode fatty acid retinoid binding protein (*far*) and thioredoxin-like protein. Interestingly, many miRNAs were predicted to target the same effector gene such as miR-50_1 and miR-252_1 target glutathione-S-transferase; while miR-86_1 and miR-252_1 target transthyretin-like protein. A few miRNAs were also predicted to target unknown secretory proteins (for example, miR-58_1 targets Minc03950, miR-9_1 targets Minc09978, and miR-7904_1 targets Minc01625, Minc07307 and Minc08734). Also, miRNAs were predicted to target other RKN genes for example, miR-7904_1 targets peptidases, tubulin, nematode cuticle collagen and UDP-glucuronosyl transferase. Interestingly, miR-58_1 was predicted to target FMRFamide-like peptide (*flp*) specifically.

We have also looked the expression profiles of RKN miRNA target genes, which were obtained from mRNA-seq data of the same set of disease development stages from which small RNA data was generated in our lab [35]. The correlation between digital expression profiles of miRNAs and their targets was studied by calculating log_2_ fold change at different stages relative to stage 1 during susceptible response. A few miRNAs showed a negative correlation with their targets at various stages, in which miRNAs were upregulated (S14 Table) and their targets were downregulated (S5 Fig). For example, miR-58_1 showed a negative correlation with FMRFamide-like peptide (*flp*) and an unknown secretory protein at stages 4 and 5. Also, a negative correlation between miR-7904_1 and two C-type lectin genes was observed at stages 3, 4 and 5. Further, miR-279_1 and miR-9_1 showed a negative correlation with sulphate anion transporter and tropomyosin, respectively at stage 5. Additionally, GO enrichment analysis was performed to understand the distribution of predicted miRNA targets under different GO terms across 19,212 protein coding genes of RKN. The results showed 46 enriched GO terms with Bonferroni corrected P-value < 0.05 (Table 2). The majority of enriched terms belong to biological processes like embryo development, nematode larval development, reproduction, growth, determination of adult span, and locomotion. Many miRNAs were found to target genes that are involved in same biological process (Table 2). For example, let-7, lin-4 and miR-92_1 target Minc04127, Minc06476 and Minc02857 genes, respectively, which are associated with embryo development (GO:0009792) process. Among these genes, Minc04127 is specifically involved in embryo development, whereas Minc06476 is involved in embryo development, nematode larval development, reproduction and growth.

**Table 2:** GO enrichment analysis of RKN miRNA targets performed with GeneMerge v1.4.

GO_ID	Pop_frac	Study_frac	Bonf_Cor_P value	Description	GO_domain
GO:0009792	2201/19212	254/1323	9.65E-15	embryo development ending in birth or egg hatching	biological_process
GO:0002119	1586/19212	196/1323	1.13E-13	nematode larval development	biological_process
GO:0000003	1625/19212	186/1323	1.14E-09	reproduction	biological_process
GO:0005861	20/19212	14/1323	1.18E-09	troponin complex	cellular_component
GO:0000166	463/19212	75/1323	2.60E-09	nucleotide binding	molecular_function
GO:0008340	681/19212	95/1323	2.40E-08	determination of adult lifespan	biological_process
GO:0040007	1298/19212	151/1323	6.15E-08	growth	biological_process
GO:0006457	81/19212	24/1323	4.80E-07	protein folding	biological_process
GO:0051015	39/19212	16/1323	1.73E-06	actin filament binding	molecular_function
GO:0008289	29/19212	13/1323	1.58E-05	lipid binding	molecular_function
GO:0005515	1719/19212	176/1323	3.08E-05	protein binding	molecular_function
GO:0040011	1003/19212	115/1323	3.11E-05	locomotion	biological_process
GO:0040035	643/19212	82/1323	3.71E-05	hermaphrodite genitalia development	biological_process
GO:0007155	36/19212	14/1323	4.06E-05	cell adhesion	biological_process
GO:0005525	179/19212	34/1323	4.97E-05	GTP binding	molecular_function
GO:0005524	802/19212	96/1323	5.92E-05	ATP binding	molecular_function
GO:0051082	44/19212	15/1323	0.000105313	unfolded protein binding	molecular_function
GO:0006937	19/19212	10/1323	0.000107934	regulation of muscle contraction	biological_process
GO:0007626	16/19212	9/1323	0.000222971	locomotory behavior	biological_process
GO:0006096	16/19212	9/1323	0.000222971	glycolytic process	biological_process
GO:0006397	30/19212	12/1323	0.000261304	mRNA processing	biological_process
GO:0003723	208/19212	35/1323	0.000680986	RNA binding	molecular_function
GO:0007264	85/19212	20/1323	0.00083496	small GTPase mediated signal transduction	biological_process
GO:0032324	5/19212	5/1323	0.001376255	molybdopterin cofactor biosynthetic process	biological_process
GO:0005200	24/19212	10/1323	0.001665741	structural constituent of cytoskeleton	molecular_function
GO:0042302	120/19212	24/1323	0.001677579	structural constituent of cuticle	molecular_function
GO:0016020	573/19212	70/1323	0.001785218	membrane	cellular_component
GO:0003774	20/19212	9/1323	0.002542324	motor activity	molecular_function
GO:0016459	20/19212	9/1323	0.002542324	myosin complex	cellular_component
GO:0004553	55/19212	15/1323	0.002654259	hydrolase activity, hydrolyzing O-glycosyl compounds	molecular_function

The 30 out of 46 enriched GO terms under three GO domains (biological Process, cellular component and molecular function) with Bonferroni corrected P-value < 0.05 are shown in table. Pop_frac describes the number of protein coding genes in the genome and study_frac describes the number of miRNA target genes in the present study with different GO terms.

## Discussion

This study reports genome-wide identification and characterization of miRNAs of both tomato and RKN from RKN-infected susceptible tomato roots at five stages of disease development under soil-grown conditions. The differential expression of miRNAs based on digital expression profiling suggests their potential role during susceptible interaction. Further, qRT-PCR results revealed that majority of tomato miRNAs were significantly upregulated at stage 3 (feeding J2s and J3s/ expansion of feeding sites). However, some of the conserved miRNAs including miR393(i), miR482(i), miR1446(i) and variant of miR156(i) and 4 novel miRNAs including Sly_miRNA667, Sly_miRNA996, Sly_miRNA1987 and Sly_miRNA2712 were also upregulated at stage 1 (invasion of J2s/ initiation of feeding sites). A few miRNAs such as miR164(i), miR319(iv) and miR1446(i) were significantly upregulated at stages 3 and 5 (J4s and females/ maintenance of feeding sites). However, miR319(iv) was significantly downregulated at stage 2 (parasitic J2s/ formation of feeding sites). Interestingly, in contrast to susceptible response, most of the miRNAs were downregulated during resistance response. The up- and down-regulation of distinct miRNAs at different stages suggest their likely role during RKN parasitism in tomato. Our report is supported by earlier studies that demonstrated the differential expression of miRNAs during plant-nematode interactions [17–20,42]. In our study, the role of both tomato and RKN miRNAs in plant-nematode interaction was investigated by identifying miRNA targets followed by their GO analysis. Among the validated tomato miRNA targets, *SBP*, *NAC*, *GAMYB-like*, *HB* and *GRAS* transcription factors, targets of conserved miRNAs, miR156(i), miR164(i), miR159(i), miR166(i) and miR171(i), respectively were enriched under GO term, transcription factor activity. Also, *MYB-like* transcription factor validated as target of novel miRNA, Sly_miRNA996, was enriched under GO term, cellular process. Furthermore, the correlation in expression profiles of tomato and RKN miRNAs with their targets was determined and discussed below.

### Tomato miRNAs involved in development of feeding cells in RKN-infected tomato roots during susceptible response

*NAC* transcription factors are known to positively regulate developmental processes such as shoot apical meristem formation, leaf and seed development and abiotic and biotic stress responses [49]. We confirmed through 5’RLM-RACE that tomato miR164(i) targets a *GOB*-like gene that encodes *NAC-*domain transcription factor. Also, miR164(i) showed a negative correlation in expression pattern with *NAC* at stages 3 and 5 based on qRT-PCR during which miR164(i) was upregulated and *NAC* was downregulated (not reported previously in tomato-RKN interaction). Earlier report has shown that miR164 mediated regulation of NAC transcription factors is required for formation of lateral organ boundaries at apical meristem and in developing compound leaves in tomato [50]. Therefore, it can be suggested that differential regulation of miR164(i) and its target, *GOB*-like gene (*NAC*-domain transcription factor) may be involved in tomato-RKN susceptible interactions.

In plants, miR156 family has several members that target different members of *SPL*/*SBP* gene family and thereby modulate plant development, including, vegetative phase transition and embryo patterning. In tomato, out of 15 *SBP* genes, 10 possess binding sites for miR156 and miR157 [51–53]. In our study, we have validated *SBP* transcription factor gene as a target of miR156 through 5’RLM-RACE. We also observed a negative correlation in the expression patterns of miR156(i) and *SBP* at stage 3 based on qRT-PCR, in which miR156(i) was upregulated and *SBP* was downregulated. Although, Zhao et al. [20] also reported an inverse correlation in expression profiles of miR156 and *SBP*, we report a negative correlation in expression between different member of miR156 and *SBP* family. This suggests that several members of miR156 family may be involved in the regulation of different members of *SPL*/*SBP* gene family during RKN pathogenesis in tomato.

By negatively regulating the expression of *GRF'*s and cell cycle genes, miR396 limits the mitotic cell division and cell proliferation during plant growth has been implicated in previous study [54]. It has also been reported that miR396/*GRFs* regulation is required for appropriate formation and maintenance of syncytium in *Arabidopsis* roots [19]. Recently, a similar result has been reported during RKN invasion in tomato roots [20]. Furthermore, as with previous report, we also observed upregulation of miR396(i) and downregulation of *GRF1* transcription factor gene during later stage 3 based on qRT-PCR. Taken together it can be concluded that the coordinated regulation of miR396 and *GRFs* plays a role in formation and maintenance of both syncytium and giant cells in nematode infected roots.

In our study, miR159(i) and miR319(iv) were upregulated in response to RKN infection based on qRT-PCR data. Furthermore, both miR159(i) and miR319(iv) were predicted to target *GAMYB-like* gene, *MYB33*. However, sequence analysis of cloned products of 5’ RLM-RACE confirmed that only miR159(i) mediates cleavage of *GAMYB-like* gene, *MYB33* in tomato roots during RKN infection and exhibited a negative correlation in expression profile at stage 3 based on qRT-PCR. A similar result has been reported previously, in which negative correlation was observed between the expression of miR159 and *MYB* transcription factor gene in RKN-infected tomato roots [20]. High expression of miR159 in vegetative tissues represses the expression of *GAMYB-like* genes to allow cell proliferation and proper plant growth and development [55,56]. The observed downregulation of *GAMYB-like* gene and upregulation of miR159(i) during tomato-RKN interaction might be required for the development of giant cells. Additionally, our study has also confirmed through 5’RLM-RACE that miR159(i) targets another *GAMYB-like*, gene *MYB65*. However, inverse correlation between miR159(i) and *GAMYB-like*, gene *MYB65* was demonstrated at stage 3 through qRT-PCR, during which the expression of *GAMYB-like* gene was higher than that of miR159(i). A similar expression profile was also observed for miR168(i) and its target *AGO1* at different stages. It is likely that these miRNAs do not regulate the expression of their respective targets in the infected tomato roots.

In our study, miR169(v) was significantly upregulated at early stages (stage 1 and 2) and late stage (stage 5) based on qRT-PCR. The highest expression was observed during stage 1 followed by stage 2. Previously, however, another member of miR169 was upregulated only at 7dpi root during *Arabidopsis*-cyst nematode infection [18]. Our study also showed that miR169(v) was downregulated at stage 2 during resistance response in contrast to susceptible response. Similarly, during soybean-cyst nematode interaction, the expression of miR169 was upregulated during susceptible response and downregulated during resistance response [42]. Known targets of miR169 are *NF* transcription factors that regulate various abiotic stress responsive genes [57]. In recent study, a different member of miR169 family was shown to be downregulated and *NF* transcription factor gene was upregulated at early stage of infection during *Arabidopsis*-RKN interaction [17]. Our results together with previous reports suggest that members of miR169 family are likely to play a role during nematode pathogenesis.

#### Characterization of a novel tomato miRNA

We identified a novel miRNA, Sly_miRNA996 that was differentially expressed at different stages of disease development. Interestingly, Sly_miRNA996 showed a negative correlation in expression with its target *MYB-like* transcription factor gene (an uncharacterized protein) at stages 1 and 3 based on qRT-PCR. The *MYB* transcription factor is a large gene family in plants involved in various processes like development, differentiation, metabolism, hormone signaling, defense, abiotic and biotic stress responses [58]. It is likely that Sly_miRNA996 by regulating *MYB-like* transcription factor plays a role during tomato-RKN interaction.

### Identification and characterization of RKN miRNAs

We identified 328 RKN miRNAs (21–24 nt length) from the same small RNAseq dataset based on the RKN genome as a reference [59]. In total, 38 conserved miRNAs, 106 novel miRNAs and 184 candidate novel miRNAs of RKN were identified. The digital expression profile of these miRNAs showed differential expression across five disease development stages suggesting their probable role during nematode parasitism. For example, conserved miRNAs including miR-100_3, miR-58_1 and lin-4 showed a notable differential expression at different stages in concordance to the previous studies [25–27]. The target prediction of RKN miRNAs provided limited data due to the lack of 3’UTRs annotation of some RKN genes. Nonetheless, the target prediction data revealed that each miRNA target many genes. For example, differentially expressed miRNAs, lin-4 and miR-100_3, target several genes involved in nucleic acid binding, protein binding, locomotion, growth, embryo development, reproduction and determination of adult lifespan. Furthermore, a differentially expressed miR-58_1 also predicted to target genes involved in gonad development, ATP binding, reproduction, embryo development, nematode larval development and determination of adult lifespan. Moreover, a previous study reported that mutants of *C*. *elegans* with deleted miR-58 family members showed abnormalities in body size, locomotion, egg laying and inability to form dauer larva [60]. Recently, FLPs (*flp* 18 and *flp* 11) were reported as specific targets of miR-58 in *C*. *elegans* [61]. In our study, FLPs were also predicted as targets of miR-58_1 and a significant negative correlation in expression between miR-58_1 and FLP was observed at stages 4 and 5 during susceptible response. FLPs are the largest family of neuropeptides in invertebrates that are responsible for nerve and muscle activity and are regulators of feeding behavior, locomotion, sensory modulation, energy balance and reproduction [62]. It is likely that miR-58_1 regulated FLPs might be playing a role during susceptible tomato-RKN interaction.

In conclusion, our comprehensive study reports the genome-wide identification and characterization of miRNAs simultaneously from both tomato and RKN during five stages of disease progression and development of parasitic nematode in RKN-infected tomato roots under soil grown conditions. In tomato, differential expression of miRNAs demonstrated through qRT-PCR in both susceptible and resistance responses indicate that host miRNAs play an important role during plant-nematode interactions. The predicted and validated targets of majority of the miRNAs were transcription factors that may be involved in regulating the genes required for giant cell development. Furthermore, negative correlation between expression levels of selected conserved and novel miRNAs and their targets was demonstrated through qRT-PCR. Additionally, differential expression of RKN miRNAs, their predicted targets and GO enrichment analysis suggest that RKN miRNAs are likely to be regulating key genes involved in nematode parasitism. Moreover, this study has identified a few novel and conserved miRNAs of both tomato [e.g., Sly_miRNA996 and miR169(v)] and RKN (e.g., miR-58_1 and miR-100_3) and their targets that can be further functionally characterized to get better insights into plant-nematode interaction. Finally, this study and previous studies strongly support for a wide range of roles of plant miRNAs during disease progression [16,17] and of RKN miRNAs in its development and parasitism during plant-nematode interactions [26]. In this context, the mobility of RNA molecules between different hosts and parasites is important, as reported in a recent review article [63]. It is likely that future work on miRNAs from both plants and parasitic nematodes would reveal their role in the regulation of each other genes (i.e., cross-talk) involved in plant defense and nematode parasitism.

## Supporting information

S1 Fig Length distribution of putative small RNA sequences obtained from all the libraries of susceptible (tomato-RKN) interaction investigated in this study. (TIF)

S2 Fig Predicted secondary structure of precursors of novel miRNAs. **(A)** Tomato miRNAs and **(B)** RKN miRNAs prepared through Mfold web server.
(TIF)

S3 Fig Genomic locus distribution of predicted miRNA precursors of tomato miRNAs. (TIF)

S4 Fig Sequence alignment of RKN miRNAs identified in our study with conserved miRNAs of other nematode species present in miRBASE v21 depository. (**A)** Sequence alignment of three RKN miRNAs (identified in our study but not reported previously) was done on the basis of same seed sequence and 80% homology within the mature miRNA sequence (21-24nt). (**B)** Sequence alignment of additional three miRNAs identified from our sequencing data when mapped on *C*. *elegans* genome with no mismatches. The nucleotides highlighted in black are same. miR- miRNA sequences of RKN identified from our sequencing data. min-miR—miRNA sequences of RKN identified by Zhang et al. 2016. MI-miR—miRNA sequences of RKN identified by Subramanian et al. 2016. cel-miR–*C*. *elegans*, prd-miR–*P*. *redivivus*, asu-miR–*A*. *suum*, bma-miR–*B*. *malayi* miRNA sequences.
(TIF)

S5 Fig A negative correlation between the digital expression profile (log_2_ fold change) of RKN miRNAs and their targets at different disease development stages relative to stage 1 is depicted in bar graph. (TIF)

S1 Table Details of primers used in qRT-PCR and 5'RLM-RACE. (XLS)

S2 Table The miRCat summary of conserved tomato miRNAs identified during tomato-RKN interaction. (XLS)

S3 Table The miRCat summary of novel tomato miRNAs identified during tomato-RKN interaction. (XLS)

S4 Table The miRCat summary of candidate novel tomato miRNAs identified during tomato-RKN interaction. (XLS)

S5 Table Digital expression profile of conserved tomato miRNAs at five disease development stages during tomato-RKN interaction. (XLS)

S6 Table Digital expression profile of novel tomato miRNAs at five disease development stages during tomato-RKN interaction. (XLS)

S7 Table Digital expression profile of candidate novel tomato miRNAs at five disease development stages during tomato-RKN interaction. (XLS)

S8 Table Target prediction of conserved tomato miRNAs through psRNAtarget analysis server. (XLS)

S9 Table Target prediction of novel tomato miRNAs through psRNAtarget analysis server. (XLS)

S10 Table Gene ontology (GO) of predicted targets of conserved and novel tomato miRNAs. (XLS)

S11 Table The miRCat miRNA summary of conserved RKN miRNAs identified during tomato-RKN interaction. (XLS)

S12 Table The miRCat miRNA summary of novel RKN miRNAs identified during tomato-RKN interaction. (XLS)

S13 Table The miRCat miRNA summary of candidate novel RKN miRNAs identified during tomato-RKN interaction. (XLS)

S14 Table Digital expression profile of conserved RKN miRNAs at five disease development stages during tomato-RKN interaction. (XLS)

S15 Table Digital expression profile of novel RKN miRNAs at five disease development stages during tomato-RKN interaction. (XLS)

S16 Table Conservation of RKN miRNAs in free-living and animal parasitic nematodes. (DOC)

S17 Table Predicted targets of conserved RKN miRNAs. (XLS)

## References

[1] Trudgill DL, Blok VC. Apomictic, polyphagous root-knot nematodes: exceptionally successful and damaging biotrophic root pathogens. Annu Rev Phytopathol. 2001; 39:53–77. 10.1146/annurev.phyto.39.1.53 11701859

[2] Castagnone-Sereno P, Danchin EG, Perfus-Barbeoch L, Abad P. Diversity and evolution of root-knot nematodes, genus *Meloidogyne*: new insights from the genomic era. Annu Rev Phytopathol. 2013; 51:203–20. 10.1146/annurev-phyto-082712-102300 23682915

[3] Williamson VM, Hussey RS. Nematode pathogenesis and resistance in plants. Plant Cell. 1996; 8:1735–1745. 10.1105/tpc.8.10.1735 8914324 PMC161311

[4] Escobar C, Barcala M, Cabrera J, Fenoll C. Overview of Root-Knot Nematodes and Giant Cells In: Escobar C, Fenoll C, editors. Advances in Botanical Research. Amsterdam: Elsevier; 2015 pp. 76:1–32.

[5] Hewezi T, Baum TJ. Manipulation of plant cells by cyst and root-knot nematode effectors. Mol Plant-Microbe Interact. 2013; 26:9–16. 10.1094/MPMI-05-12-0106-FI 22809272

[6] Kyndt T, Vieira P, Gheysen G, de Almeida-Engler J. Nematode feeding sites: unique organs in plant roots. Planta. 2013; 238:807–18. 10.1007/s00425-013-1923-z 23824525

[7] Quentin M, Abad P, Favery B. Plant parasitic nematode effectors target host defense and nuclear functions to establish feeding cells. Front Plant Sci. 2013; 4:53 10.3389/fpls.2013.00053 23493679 PMC3595553

[8] Shukla N, Kaur P, Kumar A. Molecular aspects of plant-nematode interactions. Ind J Plant Physiol. 2016; 21:477–488.

[9] Caillaud MC, Dubreuil G, Quentin M, Perfus-Barbeoch L, Lecomte P, Engler JDA, et al Root-knot nematodes manipulate plant cell functions during a compatible interaction. J Plant Physiol. 2008; 165:104–13. 10.1016/j.jplph.2007.05.007 17681399

[10] Barcala M, García A, Cabrera J, Casson S, Lindsey K, Favery B, et al Early transcriptomic events in microdissected *Arabidopsis* nematode-induced giant cells. Plant J. 2010; 61:698–712. 10.1111/j.1365-313X.2009.04098.x 20003167

[11] Portillo M, Cabrera J, Lindsey K, Topping J, Andrés MF, Emiliozzi M, et al Distinct and conserved transcriptomic changes during nematode-induced giant cell development in tomato compared with *Arabidopsis*: a functional role for gene repression. New Phytol. 2013; 197:1276–1290. 10.1111/nph.12121 23373862

[12] Jones-Rhoades MW, Bartel DP, Bartel B. MicroRNAs and their regulatory roles in plants. Annu Rev Plant Biol. 2006; 57:19–53. 10.1146/annurev.arplant.57.032905.105218 16669754

[13] Chen X. Small RNAs and their roles in plant development. Annu Rev Cell Dev Biol. 2009; 35:21–44. 10.1146/annurev.cellbio.042308.113417 PMC5135726 19575669

[14] Katiyar-Agarwal S, Jin H. Role of small RNAs in host-microbe interactions. Annu Rev Phytopathol. 2010; 48:225–246. 10.1146/annurev-phyto-073009-114457 20687832 PMC3752435

[15] Sunkar R, Li YF, Jagadeeswaran G. Functions of microRNAs in plant stress responses. Trends Plant Sci. 2012; 17:196–203. 10.1016/j.tplants.2012.01.010 22365280

[16] Hewezi T, Baum TJ. Gene silencing in nematode feeding sites In: Escobar C, Fenoll C, editors. Advances in Botanical Research. Amsterdam: Elsevier; 2015 pp. 76:221–239.

[17] Cabrera J, Barcala M, García A, Rio-Machín A, Medina C, Jaubert-Possamai S, et al Differentially expressed small RNAs in Arabidopsis galls formed by *Meloidogyne javanica*: a functional role for miR390 and its TAS3-derived tasiRNAs. New Phytol. 2016; 209:1625–1640. 10.1111/nph.13735 26542733

[18] Hewezi T, Howe P, Maier TR, Baum TJ. *Arabidopsis* small RNAs and their targets during cyst nematode parasitism. Mol Plant-Microbe Interact. 2008; 21:1622–1634. 10.1094/MPMI-21-12-1622 18986258

[19] Hewezi T, Maier TR, Nettleton D, Baum TJ. The Arabidopsis MicroRNA396-*GRF1*/*GRF3* Regulatory Module Acts as a Developmental Regulator in the Reprogramming of Root Cells during Cyst Nematode Infection. Plant Physiol. 2012; 159:321–335. 10.1104/pp.112.193649 22419826 PMC3375968

[20] Zhao W, Li Z, Fan J, Hu C, Yang R, Qi X, et al Identification of jasmonic acid-associated microRNAs and characterization of the regulatory roles of the miR319/TCP4 module under root-knot nematode stress in tomato. J Exp Bot. 2015; 66:4653–4667. 10.1093/jxb/erv238 26002970 PMC4507771

[21] Huang Y, Zou Q, Tang SM, Wang LG, Shen XJ. Computational identification and characteristics of novel microRNAs from the silkworm (Bombyx mori L.). Mol Biol Rep.2010; 37:3171–3176. 10.1007/s11033-009-9897-4 19823945

[22] Zou Q, Mao Y, Hu L, Wu Y, Ji Z. miRClassify: an advanced web server for miRNA family classification and annotation. Comput Biol Med. 2014; 45:157–160. 10.1016/j.compbiomed.2013.12.007 24480175

[23] Zeng X, Zhang X, Zou Q. Integrative approaches for predicting microRNA function and prioritizing disease-related microRNA using biological interaction networks. Brief Bioinform. 2016; 17:193–203. 10.1093/bib/bbv033 26059461

[24] Guo L, Liang T, Yu J, Zou Q. A Comprehensive Analysis of miRNA/isomiR Expression with Gender Difference. PLoS ONE. 2016; 11:p.e0154955 10.1371/journal.pone.0154955 27167065 PMC4864079

[25] Wang Y, Mao Z, Yan J, Cheng X, Liu F, Xiao L, et al Identification of microRNAs in *Meloidogyne incognita* using deep sequencing. PLoS ONE. 2015; 10:e0133491 10.1371/journal.pone.0133491 26241472 PMC4524723

[26] Zhang Y, Wang Y, Xie F, Li C, Zhang B, Nichols R L, et al Identification and characterization of microRNAs in the plant parasitic root-knot nematode *Meloidogyne incognita* using deep sequencing. Funct Integr Genomics. 2016; 16:127–142. 10.1007/s10142-015-0472-x 26743520

[27] Subramanian P, Choi IC, Mani V, Park J, Subramaniyam S, Choi KH, et al Stage-wise identification and analysis of miRNA from root-knot nematode *Meloidogyne incognita*. Int J Mol Sci. 2016; 10.3390/ijms17101758 PMC5085782 27775666

[28] Bybd DW Jr, Kirkpatrick T, Barker KR. An improved technique for clearing and staining plant tissue for detection of nematodes. J Nematol. 1983; 15:142–143. 19295781 PMC2618249

[29] Stocks MB, Moxon S, Mapleson D, Woolfenden HC, Mohorianu I, Folkes L, et al The UEA sRNA workbench: a suite of tools for analysing and visualizing next generation sequencing microRNA and small RNA datasets. Bioinformatics. 2012; 28:2059–2061. 10.1093/bioinformatics/bts311 22628521 PMC3400958

[30] Tomato Genome Consortium. The tomato genome sequence provides insights into fleshy fruit evolution. Nature. 2012; 485:635–641. 10.1038/nature11119 22660326 PMC3378239

[31] Gruber AR, Lorenz R, Bernhart SH, Neubock R, Hofacker IL. The Vienna RNA websuite. Nucleic Acids Res. 2008; 36:W70–W74. 10.1093/nar/gkn188 18424795 PMC2447809

[32] Meyers BC, Axtell MJ, Bartel B, Bartel DP, Baulcombe D, Bowman JL, et al Criteria for Annotation of Plant MicroRNAs. Plant Cell. 2008; 20:3186–3190. 10.1105/tpc.108.064311 19074682 PMC2630443

[33] Kozomara A, Griffiths-Jones S. miRBase: integrating microRNA annotation and deep-sequencing data. Nucleic Acids Res. 2011; 39:D152–D157. 10.1093/nar/gkq1027 21037258 PMC3013655

[34] Dai X, Zhao PX. psRNATarget: a plant small RNA target analysis server. Nucleic Acids Res. 2011; 39:155–159. 21622958 10.1093/nar/gkr319 PMC3125753

[35] Shukla N, Yadav R, Kaur P, Rasmussen S, Goel S, Agarwal M, et al Transcriptome analysis of root-knot nematode (*Meloidogyne incognita*)-infected tomato (*Solanum lycopersicum*) roots reveals complex gene expression profiles and metabolic networks of both host and nematode during susceptible and resistance responses. Mol Plant Pathol. 2017; 10.1111/mpp.12547 PMC6638136 28220591

[36] Du Z, Zhou X, Ling Y, Zhang Z, Su Z. AgriGO: a GO analysis toolkit for the agricultural community. Nucleic Acids Res. 2010; 38:64–70. 10.1093/nar/gkq310 PMC2896167 20435677

[37] Livak KJ, Schmittgen TD. Analysis of relative gene expression data using real-time quantitative PCR and the 2^–ΔΔCT^ method. Methods. 2001; 25:402–408. 10.1006/meth.2001.1262 11846609

[38] Betel D, Koppal A, Agius P, Sander C, Leslie C. Comprehensive modeling of microRNA targets predicts functional non-conserved and non-canonical sites. Genome Biol. 2010; 11:R90 10.1186/gb-2010-11-8-r90 20799968 PMC2945792

[39] Castillo-davis CI, Hartl DL. GeneMerge-post-genomic analysis, data mining, and hypothesis testing. Bioinformatics. 2003; 19:891–892. 12724301 10.1093/bioinformatics/btg114

[40] Axtell MJ. Classification and Comparison of Small RNAs from Plants. Annu Rev Plant Biol. 2013; 64:137–159. 10.1146/annurev-arplant-050312-120043 23330790

[41] Song C, Wang C, Zhang C, Korir NK, Yu H, Ma Z, et al Deep sequencing discovery of novel and conserved microRNAs in trifoliate orange (*Citrus trifoliata*). BMC Genomics. 2010; 11:431–442. 10.1186/1471-2164-11-431 20626894 PMC2996959

[42] Li X, Wang X, Zhang S, Liu D, Duan Y, Dong W. Identification of Soybean MicroRNAs Involved in Soybean Cyst Nematode Infection by Deep Sequencing. PLoS ONE. 2012; 7:e39650 10.1371/journal.pone.0039650 22802924 PMC3384596

[43] Lakhotia N, Joshi G, Bhardwaj AR, Katiyar-Agarwal S, Agarwal M, Jagannath A, et al Identification and characterization of miRNAome in root, stem, leaf and tuber developmental stages of potato (*Solanum tuberosum* L.) by high-throughput sequencing. BMC Plant Biol. 2014;14:6 10.1186/1471-2229-14-6 24397411 PMC3913621

[44] Zhang B, Pan X, Cannon CH, Cobb GP, Anderson TA. Conservation and divergence of plant microRNA genes. Plant J. 2006; 46:243–259. 10.1111/j.1365-313X.2006.02697.x 16623887

[45] Voinnet O. Origin, Biogenesis, and Activity of Plant MicroRNAs. Cell. 2009; 136:669–687. 10.1016/j.cell.2009.01.046 19239888

[46] Li A, Mao L. Evolution of plant microRNA gene families. Cell Research. 2007; 17:212–218. 10.1038/sj.cr.7310113 17130846

[47] Chen L, Ren Y, Zhang Y, Xu J, Zhang Z, Wang Y. Genome-wide profiling of novel and conserved *Populus* microRNAs involved in pathogen stress response by deep sequencing. Planta. 2012; 235:873–883. 10.1007/s00425-011-1548-z 22101925

[48] C. elegans Sequencing Consortium. Genome sequence of the nematode *C*. *elegans*: A platform for investigating biology. Science. 1998; 282:2012–2018. 9851916 10.1126/science.282.5396.2012

[49] Nuruzzaman M, Sharoni AM, Kikuchi S. Roles of NAC transcription factors in the regulation of biotic and abiotic stress responses in plants. Front Microbiol. 2013; 4:248 10.3389/fmicb.2013.00248 24058359 PMC3759801

[50] Berger Y, Harpaz-Saad S, Brand A, Melnik H, Sirding N, Alvarez JP, et al The NAC-domain transcription factor GOBLET specifies leaflet boundaries in compound tomato leaves. Development. 2009; 136:823–832. 10.1242/dev.031625 19176589

[51] Moxon S, Jing R, Szittya G, Schwach F, Rusholme Pilcher RL, Moulton V, et al Deep sequencing of tomato short RNAs identifies microRNAs targeting genes involved in fruit ripening. Genome Res. 2008; 18:1602–1609. 10.1101/gr.080127.108 18653800 PMC2556272

[52] Salinas M, Xing S, Hoehmann S, Berndtgen R, Huijser P. Genomic organization, phylogenetic comparison and differential expression of the SBP-box family of transcription factors in tomato. Planta. 2012; 235:1171–1184. 10.1007/s00425-011-1565-y 22160465

[53] Karlova R, van Haarst JC, Maliepaard C, van de Geest H, Bovy AG, Lammers M, et al Identification of microRNA targets in tomato fruit development using high-throughput sequencing and degradome analysis. J Exp Bot. 2013; 64:1863–1878. 10.1093/jxb/ert049 23487304 PMC3638818

[54] Rodriguez RE, Mecchia MA, Debernardi JM, Schommer C, Weigel D, Palatnik JF. Control of cell proliferation in *Arabidopsis thaliana* by microRNA miR396. Development. 2010; 137:103–112. 10.1242/dev.043067 20023165 PMC2796936

[55] Allen RS, Li J, Stahle MI, Dubrou’e A, Gubler F, Millar AA. Genetic analysis reveals functional redundancy and the major target genes of the *Arabidopsis* miR159 family. Proc Natl Acad Sci. USA. 2007; 104:16371–16376. 10.1073/pnas.0707653104 17916625 PMC2042213

[56] Alonso-Peral MM, Li J, Li Y, Allen RS, Schnippenkoetter W, Ohms S, et al The MicroRNA159-Regulated *GAMYB-like* Genes Inhibit Growth and Promote Programmed Cell Death in Arabidopsis. Plant Physiol. 2010; 154:757–771. 10.1104/pp.110.160630 20699403 PMC2949021

[57] Khraiwesh B, Zhu JK, Zhu J. Role of miRNAs and siRNAs in biotic and abiotic stress responses of plants. Biochim Biophys Acta. 2012; 1819:137–148. 10.1016/j.bbagrm.2011.05.001 21605713 PMC3175014

[58] Ambawat S, Sharma P, Yadav NR, Yadav RC. MYB transcription factor genes as regulators for plant responses: an overview. Physiol Mol Biol Plants. 2013; 19:307–321. 10.1007/s12298-013-0179-1 24431500 PMC3715649

[59] Abad P, Gouzy J, Aury J-M, Castagnone-Sereno P, Danchin EGJ, Deleury E, et al Genome sequence of the metazoan plant-parasitic nematode *Meloidogyne incognita*. Nat Biotechnol. 2008; 26:909–915. 10.1038/nbt.1482 18660804

[60] Alvarez-saavedra E, Horvitz HR. Many families of *Caenorhabditis elegans* microRNAs are not essential for development or viability. Curr Biol. 2010; 20:367–373. 10.1016/j.cub.2009.12.051 20096582 PMC2844791

[61] Subasic D, Brümmer A, Wu Y, Pinto SM, Imig J, Keller M, et al Cooperative target mRNA destabilization and translation inhibition by miR-58 microRNA family in *C*. *elegans*. Genome Res. 2015; 25: 1680–1691. 10.1101/gr.183160.114 26232411 PMC4617964

[62] Peymen K, Watteyne J, Frooninckx L, Schoofs L, Beets I. The FMRFamide-like peptide family in nematodes. Front Endocrinol. 2014; 10.3389/fendo.2014.00090 PMC4058706 24982652

[63] Knip M, Constantin ME, Thordal-Christensen H. Trans-kingdom Cross-Talk: Small RNAs on the Move. 2014; PLoS Genet. 10: e1004602 10.1371/journal.pgen.1004602 25188222 PMC4154666

