# Supplementary figures and images for: Genome-wide identification and characterization of miRNAome from tomato (Solanum lycopersicum) roots and root-knot nematode (Meloidogyne incognita) during susceptible interaction
Source: PLoS One. 2017 Apr 20;12(4):e0175178. doi: 10.1371/journal.pone.0175178 (PMC5398497; doi:10.1371/journal.pone.0175178)

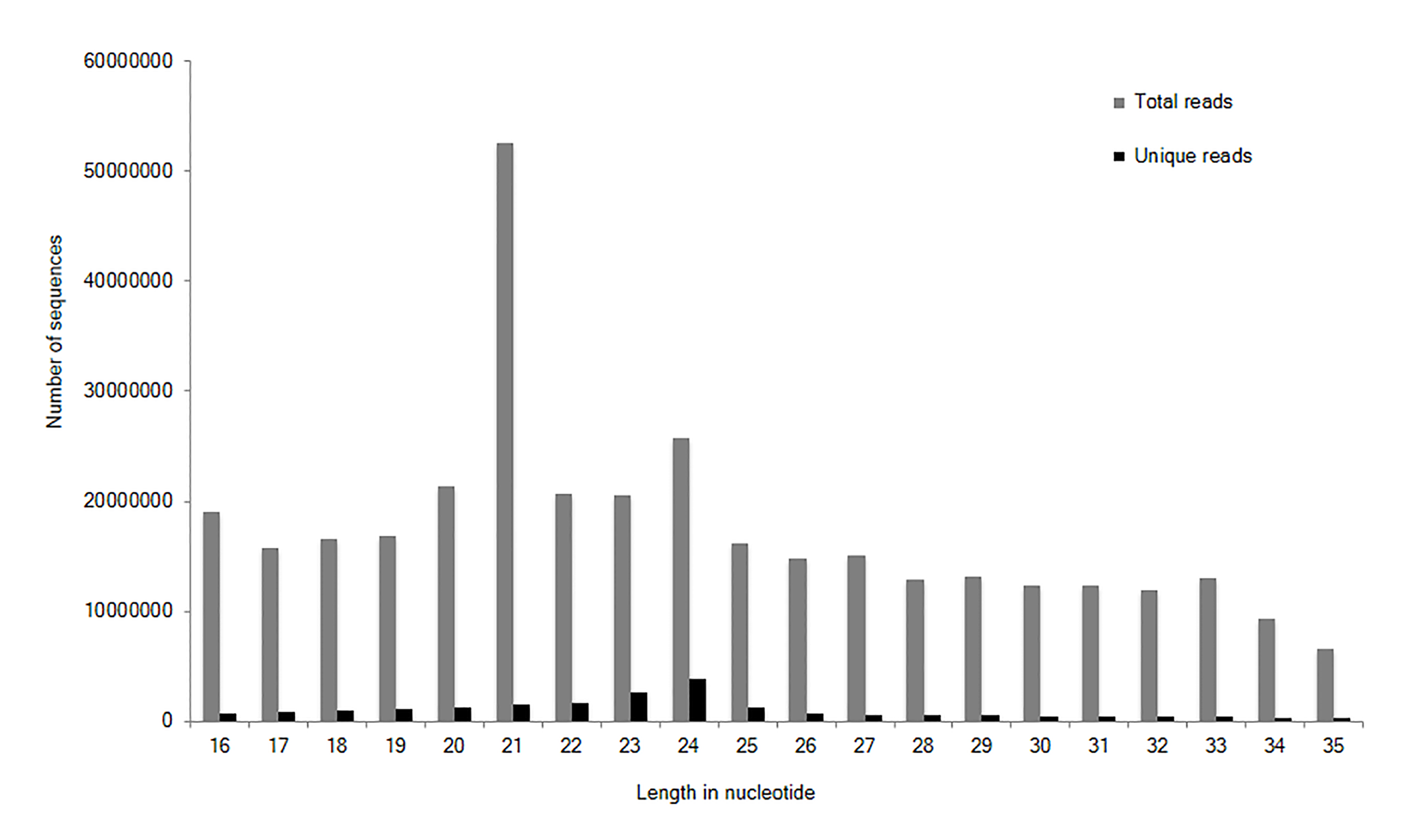

Supplement: S1 Fig — (TIF) [file pone.0175178.s001.tif]

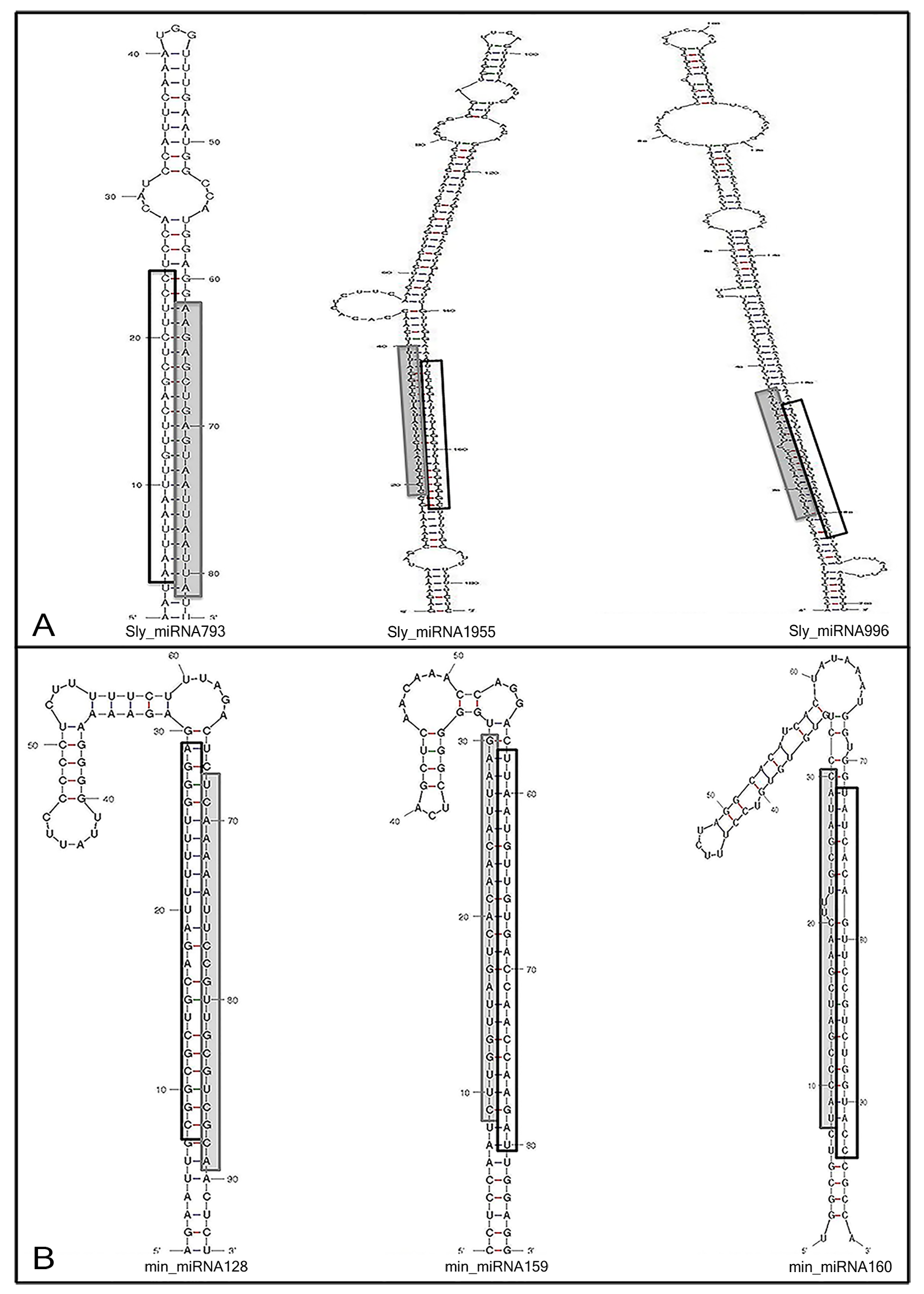

Supplement: S2 Fig — (A) Tomato miRNAs and (B) RKN miRNAs prepared through Mfold web server. (TIF) [file pone.0175178.s002.tif]

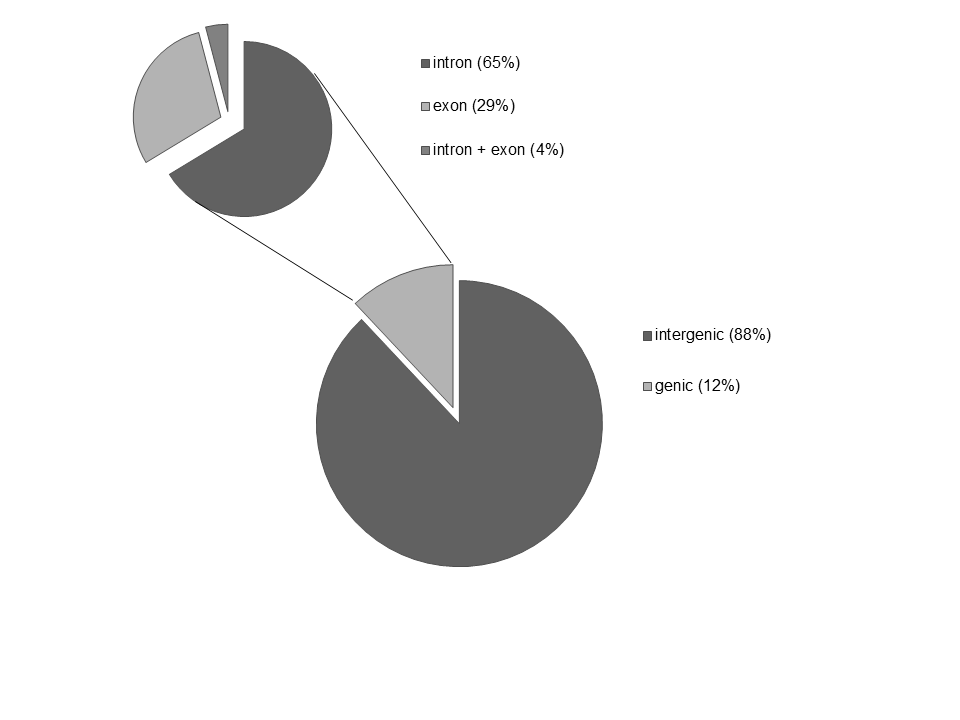

Supplement: S3 Fig — (TIF) [file pone.0175178.s003.tif]

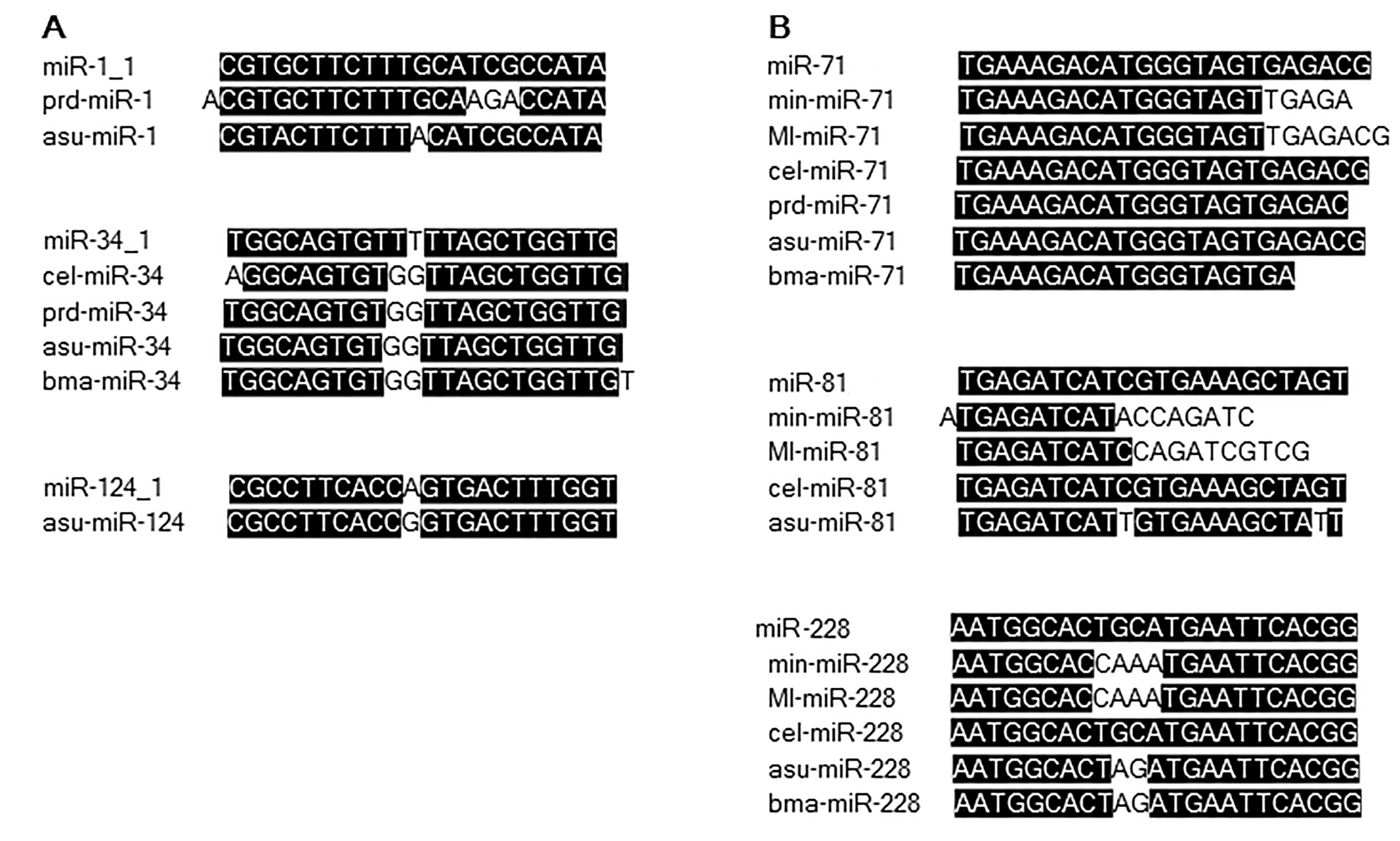

Supplement: S4 Fig — (A) Sequence alignment of three RKN miRNAs (identified in our study but not reported previously) was done on the basis of same seed sequence and 80% homology within the mature miRNA sequence (21-24nt). (B) Sequence alignment of additional three miRNAs identified from our sequencing data when mapped on C. elegans genome with no mismatches. The nucleotides highlighted in black are same. miR- miRNA sequences of RKN identified from our sequencing data. min-miR—miRNA sequences of RKN identified by Zhang et al. 2016. MI-miR—miRNA sequences of RKN identified by Subramanian et al. 2016. cel-miR–C. elegans, prd-miR–P. redivivus, asu-miR–A. suum, bma-miR–B. malayi miRNA sequences. (TIF) [file pone.0175178.s004.tif]

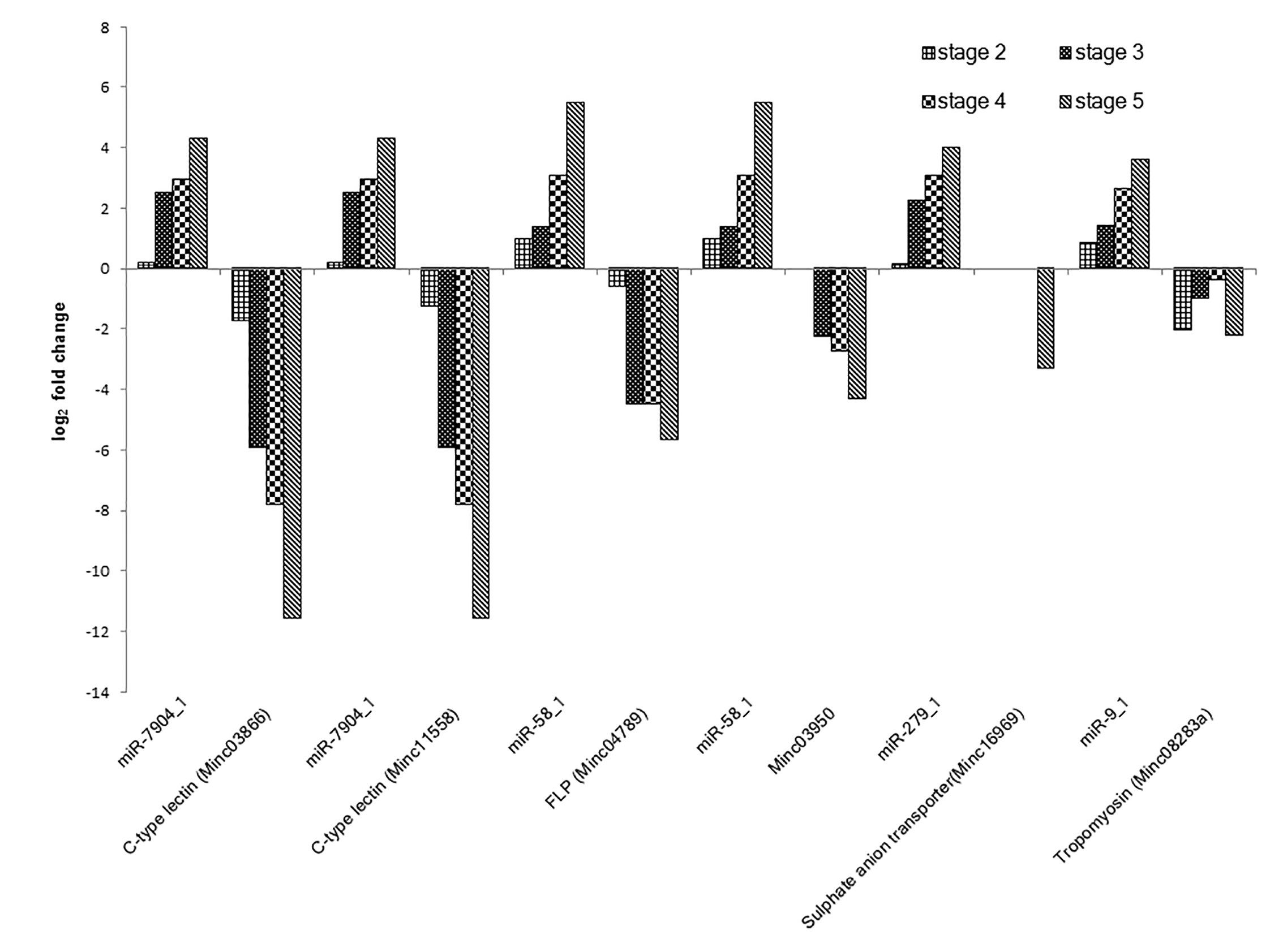

Supplement: S5 Fig — (TIF) [file pone.0175178.s005.tif]
