# Supplementary material for: Genome-wide identification and characterization of miRNAome from tomato (Solanum lycopersicum) roots and root-knot nematode (Meloidogyne incognita) during susceptible interaction
Source: PLoS One. 2017 Apr 20;12(4):e0175178. doi: 10.1371/journal.pone.0175178 (PMC5398497; doi:10.1371/journal.pone.0175178)
Supplement: S16 Table — (DOC) [file pone.0175178.s021.doc]

**S16 Table. Conservation of RKN miRNAs in free-living and animal parasitic nematodes. miRNA^1^ indicates conserved miRNAs identified in our study, miRNA sequence^2^ indicates sequences of conserved miRNAs identified in our study, ‘+’ in min^3^ indicates similar *M. incognita* miRNA sequences identified by Wang et al. (2015) and Zhang et al. (2016). ‘+’ in cel^4^, prd^5^, bma^6^ and asu^7^ indicates the conservation of predicted miRNAs on the basis of same seed sequence (2-7 nt from 5’ end) and 80% sequence homology within the mature miRNA sequence (21-24 nt) of *C. elegans*, *P. redivivus*, *B. malayi* and *A. suum,* respectively.**

| **miRNA^1^** | **miRNA sequence^2^** | **min^3^** | **cel^4^** | **prd^5^** | **bma^6^** | **asu^7^** |
| --- | --- | --- | --- | --- | --- | --- |
| miR-1_1 | CGTGCTTCTTTGCATCGCCATA |  |  | **+** |  | **+** |
| miR-2_1 | TATCACAGTTCGATATGGCCTC | **+** |  |  |  | **+** |
| miR-9_1 | ATAAAGCTAGATTACCAGAGCT | **+** |  | **+** | **+** | **+** |
| miR-34_1 | TGGCAGTGTTTTTAGCTGGTTG |  | **+** | **+** | **+** | **+** |
| miR-39_1 | TCACCGGGAATTTATTCATGAGT | **+** |  | **+** |  |  |
| miR-50_1 | TGATATGTCTTGTATTCTTGGGT | **+** | **+** | **+** | **+** | **+** |
| miR-57_1 | TACCCTGTAGTCCCGAGCCGTTTG |  |  |  | **+** | **+** |
| miR-58_1 | TGAGATCAGTCCAGATTCGTCG | **+** |  | **+** |  |  |
| miR-72_1 | AGGCAAGATGTTGGCATTGCTG | **+** | **+** | **+** | **+** | **+** |
| miR-76_1 | TTCGTTGTTTCTGAAACCTGAAC | **+** |  | **+** |  | **+** |
| miR-86_1 | TAAGTGAATATCTTGCCACAAGCT | **+** |  | **+** | **+** | **+** |
| miR-92_1 | TATTGCACTCGTTTCGGCCTGA | **+** |  |  | **+** | **+** |
| miR-100_1 | TACCCGTAGATCCGAACTAGTCT | **+** |  | **+** | **+** | **+** |
| miR-124_1 | CGCCTTCACCAGTGACTTTGGT |  |  |  |  | **+** |
| miR-124_2 | TAAGGCACGCGGTGAATGCTGA | **+** | **+** | **+** | **+** | **+** |
| miR-184_1 | TGGACGGAAGTCTGATAAGGAGC | **+** |  |  |  | **+** |
| miR-252_1 | CTAAGTAGTAGTGCCGCATTTA | **+** | **+** | **+** | **+** | **+** |
| miR-277_1 | TAAATGCACCTATCTGGTATGA | **+** |  | **+** |  |  |
| miR-279_1 | TGACTAGATCCACACTCATCT | **+** |  |  | **+** | **+** |
| miR-993_1 | TAAGCTCGCTTCTACAGGCGT | **+** |  | **+** |  | **+** |
| miR-7904_1 | TCAAAAATTCCGTTGCGTCGCA | **+** |  | **+** |  |  |
| let-7 | TGAGGTAGTAGGTTGTATAGTT | **+** | **+** | **+** | **+** | **+** |
| lin 4 | TCCCTGAGACTATAACTGTGA | **+** | **+** | **+** |  | **+** |
